# Evidence-based clinical practice guidelines for rapidly progressive glomerulonephritis 2014

**DOI:** 10.1007/s10157-015-1218-8

**Published:** 2016-04-21

**Authors:** Yoshihiro Arimura, Eri Muso, Shoichi Fujimoto, Midori Hasegawa, Shinya Kaname, Joichi Usui, Toshiko Ihara, Masaki Kobayashi, Mitsuyo Itabashi, Kiyoki Kitagawa, Junichi Hirahashi, Kenjiro Kimura, Seiichi Matsuo

**Affiliations:** Kyorin University, Tokyo, Japan; Kitano Hospital, Osaka, Japan; Miyazaki University, Miyazaki, Japan; Fujita Health University, Aichi, Japan; Tsukuba University, Ibaraki, Japan; Kyoto University, Kyoto, Japan; Tokyo Medical University Ibaraki Medical Center, Ibaraki, Japan; Tokyo Women’s Medical University, Tokyo, Japan; Kanazawa Medical Center, Ishikawa, Japan; Keio University, Tokyo, Japan; St. Marianna University, Kanagawa, Japan; Nagoya University, Nagoya, Japan

## Preface

### 1. Background of this guideline

Rapidly progressive glomerulonephritis (RPGN) is defined in Japan as “a syndrome that progresses rapidly within a few weeks or months to renal failure and is accompanied by urinary findings of nephritis.” The clinical concept of RPGN includes various renal diseases that cause renal function to deteriorate over a subacute course. Necrotizing crescentic glomerulonephritis is often observed in histopathological findings.

In 2002, a joint committee formed by JSN and a research group on progressive renal disorders from the specific disease program of the Ministry of Health, Labour, and Welfare released Japan’s first “Clinical Guidelines for Rapidly Progressive Glomerulonephritis.” These landmark guidelines were based on the results of research conducted overseas and a national survey on RPGN and took the particular characteristics of Japan into consideration. The RPGN guidelines were divided into diagnostic guidelines for early discovery and guidelines for making definitive diagnoses. RPGN was categorized into either a myeloperoxidase (MPO-ANCA) or proteinase-3 antineutrophil cytoplasmic (PR3-ANCA) type based on ANCA-related vasculitis. Furthermore, a practical therapeutic algorithm was created for MPO-ANCA types that took into consideration factors such as clinical severity, age, and presence of dialysis. Treatment guidelines for anti-GBM antibody RPGN were also presented. These guidelines were widely used in Japan and contributed greatly to improving RPGN prognosis.

These guidelines were revised 9 years later, in 2011, and published as “Clinical Guidelines for Rapidly Progressive Glomerulonephritis—2nd edition.” This edition took into account medical advances that had occurred since 2002, and eGFR, not serum creatinine level, was adopted for diagnosing RPGN. Moreover, MPO-ANCA RPGN and PR3-ANCA RPGN were combined under ANCA-positive RPGN. The new edition also included concise statements for treatments and dealing with complications.

Since then, marked progress has been made in RPGN research both in Japan and overseas. Globally, kidney disease improving global outcomes (KDIGO) released clinical guidelines for glomerulonephritis (“pauci-immune focal and segmental necrotizing glomerulonephritis,” “anti-GBM antibody glomerulonephritis,” and “lupus nephritis” were addressed as diseases that present with RPGN, and treatment guidelines with recommendation levels were given). In 2012, the American College of Rheumatology and European League Against Rheumatism and European Renal Association-European Dialysis and Transplant Association (EULAR/ERA-EDTA) published guidelines for lupus nephritis. There was also the 2012 Revised International Chapel Hill Consensus Conference Nomenclature of Vasculitides, which changed the names of vasculitis diseases and performed other tasks. In Japan, the biological drug rituximab for ANCA-related vasculitis (microscopic polyangiitis, granulomatosis with polyangiitis) became eligible for health insurance coverage in 2013. Against this background, JNS and a research group on progressive renal disorders from the Ministry of Health, Labour, and Welfare decided to create the “2014 RPGN Clinical Guidelines Based on Evidence.” A working group was formed to draft the guidelines.

### 2. The intended purpose, anticipated users, and predicted social significance of the guidelines

The objective of these guidelines is to present evidence-based clinical guidelines that reflect the conditions in Japan. The text was created in the format of answers to CQ that nephrologists have when treating RPGN in everyday practice. Each answer comes in the form of a statement, and statements related to treatment are given recommendation grades based on the level of evidence. The first part is in a text format and describes areas that include the definition, concept, classification, epidemiology, diagnosis, and pathology of RPGN. Data from Japan are presented in figures and tables. These guidelines are not intended to serve as a comprehensive textbook but rather to answer nephrologists’ questions and provide information on standard medical care to aid clinical judgments. For this reason, the RPGN clinical guidelines working group independently evaluated the related evidence and presented applicability criteria for therapeutic interventions, with the goals of suppressing the advance of renal dysfunction and improving survival prognosis.

Evidence from the literature can provide information but is no substitute for the specialized skills and experiences of individual physicians. Whether a particular statement applies and how it applies to a particular patient depends on the specialist abilities of each physician. The times demand that medical care shift from a one-size-fits-all approach to a tailor-made approach. Clinical guidelines are not supposed to impose a uniform style of care on physicians. Each physician needs to determine what kind of care each patient needs, based on an understanding of the content of clinical guidelines. As such, these guidelines are not intended to limit physicians to certain forms of medical behavior but were created to assist them in exercising their discretion to decide the type of care to be provided. In addition, it should be stated clearly that these guidelines are not criteria for deciding physician–patient conflicts or medical malpractice lawsuits.

### 3. Patients within the scope of the guidelines

In clinical practice, RPGN encompasses a wide range of renal diseases such as ANCA-positive RPGN, anti-GBM antibody RPGN, proliferative lupus nephritis, IgA nephropathy, and forms of immune complex RPGN such as purpura nephritis, as well as infection-associated RPGN, acute interstitial nephritis, and thrombotic microangiopathy. As each of these has different prognoses and treatment strategies, it is not possible to encompass all the diseases. These guidelines focus on ANCA-positive RPGN, which appears frequently and for which there is relatively strong evidence, and on addressing the severe primary diseases, namely lupus nephritis and anti-GBM antibody RPGN. Treatment strategies with recommendation grades are presented for each of these diseases. There is little evidence for other forms of RPGN, so these are merely mentioned in the text. These guidelines apply to RPGN patients of all ages. Finally, pregnancy-related items were, as a rule, not included.

### 4. Preparation procedure

Creating evidence-based guidelines first requires the enormous task of gathering and evaluating evidence. We would like to sincerely thank the members of the RPGN Clinical Guidelines Working Group for their dedication and effort (show list of contributors).

The first meeting of the clinical guidelines working group was held on September 23, 2011. The group was led by Dr. Kenjiro Kimura of the St. Marianna University School of Medicine, who explained the significance of creating the guidelines and the procedures for the task.

The working group then met three more times, submitting on August 24, 2012, a table of contents and a draft of the CQ. The RPGN clinical guidelines committee met on August 25, 2012 for the first time as the working group for drafting the guidelines. This was essentially considered the startup meeting. From then on, the working group began drafting the guidelines based on a shared understanding. The MINDS handbook for creating clinical guidelines was followed, and the Delphi method was used in composing CQ, which is the core of the guidelines. Recommendation grades were determined by an informal consensus. As a rule, PubMed records up to July 2012 were used to search the literature. If necessary, important studies from after this date were included, with reasons given.

Several meetings of the RPGN clinical guidelines committee were held (including review discussions among committee members through e-mail). Through this process, the initial CQ and text items were appropriately revised, and a few deletions and additions were made. The algorithm was also repeatedly revised to make the guidelines easier to use. From September 13 to October 13, 2013, each part was reviewed by two designated referees and two designated academic societies. Simultaneously, public comments were solicited from members of the Japanese Society of Nephrology (JSN). The manuscript was then revised based on the referees’ opinions and public comments. The RPGN clinical guidelines committee met on January 26, 2014, to examine the revised manuscript. Afterward, additional revisions were made as needed until a final draft was obtained. The guidelines, as well as responses to the referees’ opinions and public comments, were posted on the JSN Web site.

### 5. Contents of the guideline

The guidelines comprise the following chapters: I. Disease concepts and definitions, II. Diagnosis, III. Epidemiology and prognosis, IV. Algorithms, and V. Diagnostic and treatment CQ. Chapters I to III and the section on the side effects of immunosuppressant therapy and the methods of treating these effects are in text format. Chapter IV-2 contains 20 CQ on particularly problematic areas of everyday care. The answers to these come in the form of statements and are accompanied by recommendation grades. The evidence and background for the recommended treatments are explained in the commentary, which should be referenced as needed. The algorithms of chapter IV-1 are presented in flowcharts for diagnosis and treatment, which were created so the location of the CQ can be easily determined. Note that these guidelines were created in tandem with the “2013 CKD clinical guideline based on evidence,” and so were written by the same authors.

### 6. Evidence levels and recommendation grades

Evidence levels were evaluated in a manner similar to that described in the “2013 CKD clinical guideline based on evidence.”

- [Evidence Levels]
- Level 1: Systematic review/meta-analysis.
- Level 2: At least 1 randomized controlled trial (RCT).
- Level 3: A non-RCT.
- Level 4: An analytical epidemiologic study (cohort study or case–control study) or a single-arm intervention study (no controls).
- Level 5: A descriptive study (case report or case series).
- Level 6: Opinion of an expert committee or an individual expert, which is not based on patient data.

Evidence levels for meta-analyses and systematic reviews were determined from the designs of the studies on which they were based. If the underlying studies had mixed designs, consensus was reached to adhere to the lowest level (e.g., a meta-analysis of cohort studies would be level 4, as would a meta-analysis that included both RCT and cohort studies).

Consensus was also reached to assign evidence level 4 to all RCT subanalyses and post hoc analyses. Therefore, an RCT with a clear primary outcome would be considered level 2, while a subanalysis or post hoc analysis of this RCT would be considered level 4.

The following recommendation grades were assigned to statements about treatments, which were based on the level of evidence for each statement.

- [Recommendation Grades]
- Grade A: Strongly recommended because the scientific basis is strong.
- Grade B: Recommended because there is some scientific basis.
- Grade C1: Recommended despite having only a weak scientific basis.
- Grade C2: Not recommended because there is only a weak scientific basis.
- Grade D: Not recommended because scientific evidence shows treatment to be ineffective or harmful.

As a rule, standard treatments in Japan were recommended, but eligibility for health insurance coverage was not necessarily required. Drugs ineligible for insurance coverage were denoted as such. Recommendation grades were assigned to statements about treatment-related CQ. In addition, questions such as “To which subgroup would this be recommended?” and “To which subgroup would this not be recommended?” were addressed whenever possible. Recommendation grades were decided through consultations among the working group members by considering the tradeoffs between and balance of benefits, damage, side effects, and risk. If differing views existed among the referees or in the public comments, the group reexamined the area through an exchange of opinions. The reasons for choosing a recommendation grade and the decision-making process involved were described in the commentary, as a rule.

### 7. Issues on the preparation of this guideline

Although evidence regarding renal diseases that present with RPGN is gradually increasing in Japan, it is still insufficient, which means that these guidelines were heavily influenced by evidence from Europe and the United States. Whether the results of clinical research from the West can be applied as is to Japan is a question that deserves careful consideration. Even in the West, only a few large clinical studies on RPGN have been conducted, so the quality of evidence is limited. In creating the guidelines, we strove to ensure they would not deviate greatly from clinical practice in Japan.

The guidelines were made to be used by nephrologists. Furthermore, although there have been calls recently for clinical guidelines to address the viewpoint of patients and provide information on medical economics, these areas were not taken into consideration.

### 8. Financial sources and conflict of interest

The funds used in creating the guidelines were provided by a research group on progressive kidney disorders funded by the Ministry of Health, Labour, and Welfare’s research project for overcoming intractable diseases. These funds were used to pay for transportation to and from meetings, to rent space for meetings, and for box lunches and snacks. The committee members received no compensation. Everyone involved in creating the guidelines (including referees) submitted conflict-of-interest statements based on academic society rules, which are managed by JSN. Opinions were sought from multiple referees and related academic societies to prevent the guidelines from being influenced by any conflicts of interest. Drafts were shown to the society members, and revisions were made based on their opinions (public comments).

### 9. Publication and future revisions

The guidelines are to be published in Japanese-language journal of JNS and concurrently released in book form by Tokyo Igakusha. This guideline was also uploaded to the homepage of the JSN. They will also be posted on the MINDS Web site of the Japan Council for Quality Health Care.

It will also be necessary to verify the extent to which these guidelines are being implemented and complied with, particularly for treatments of recommendation grade B. We hope to form a new working group on RPGN to follow up on compliance under a Ministry of Health, Labour, and Welfare research group. In addition, we want to extract and organize the various research questions that came up while creating these guidelines so that new clinical research (particularly prospective interventional studies) and basic research can be conducted. We intend to participate in structuring further evidence that is accumulated on RPGN for rituximab and other new therapies. At the same time, by continuing to collect evidence regarding RPGN overall, we hope to work toward a revision of these guidelines several years from now. We will also study how to address in the next guidelines the viewpoint of patients and medical economics, which were not mentioned this time. In the future, guidelines for patients also need to be considered.

## I. Disease entity · definition (pathogenesis · pathophysiology)

The World Health Organization defines rapidly progressive glomerulonephritis (RPGN)/rapidly progressive nephritic syndrome as an abrupt or insidious onset of macroscopic hematuria, proteinuria, anemia, and rapidly progressing renal failure. The Research Committee of Progressive Glomerular Disease of the Ministry of Health, Labor and Welfare of Japan and the Japanese Society of Nephrology defined RPGN as rapidly progressing renal failure within several weeks to several months that is associated with urinary findings such as proteinuria, hematuria, red blood cell casts, and granular casts indicating glomerulonephritis. Without treatment, most patients will develop end-stage renal disease. RPGN is one of the clinical syndromes resulting from glomerulonephritis. In most cases of RPGN, the histopathological diagnosis is necrotizing crescentic glomerulonephritis (NCGN). NCGN is classified into three types—linear, granular, and paucity-immune pattern—based on immunofluorescence microscopic findings. A linear pattern indicates anti-glomerular basement disease, including in situ immune complex formation disease based on the Chapel Hill consensus criteria (2012). Granular staining is seen in circulating immune complex diseases such as systemic lupus erythematosus and IgA vasculitis. Most cases with the paucity-immune pattern are glomerulonephritis induced by antineutrophil cytoplasmic autoantibody (ANCA)-associated vasculitis. Myeloperoxidase (MPO)-specific ANCA-associated vasculitis is more widely known than proteinase 3 ANCA-associated vasculitis in Japan.

## II. Diagnosis (symptoms and signs)

General fatigue, slight fever, appetite loss, flu-like symptoms, and abnormal body weight loss are also frequently observed. Microscopic, or occasionally macroscopic, hematuria is observed accompanied by dysmorphism of red blood cells and cellular cast formation. Proteinuria is frequently present; however, nephrotic syndrome accompanying systemic edema is rare. Recently, asymptomatic cases found through urinary screening during sporadic health checks are increasing. When the causative disease of RPGN is systemic (vasculitis, systemic lupus erythematosus, etc.), a variety of extrarenal symptoms are observed, such as disorders of the upper respiratory tract, lung (pulmonary bleeding, interstitial pneumonitis), skin (purpura, erythema), digestive organ (melena, abdominal pain), or neurons.

In blood chemistry tests, elevation of serum creatinine, decrease of estimated glomerular filtration rate, and elevation of C-reactive protein and erythrocyte sedimentation rate, often refractory to treatment by antibiotics, are observed. Rapidly progressive anemia, gradual elevation of neutrophil-dominant white blood cells, and thrombocytes are frequently observed. Complement levels tend to be elevated in RPGN because of systemic vasculitis; in contrast, systemic lupus erythematosus (SLE) decreases complement levels. As autoantibodies for detecting the causative disease of RPGN, anti-glomerular basement membrane (GBM) antibody, ANCA, and anti-dsDNA antibody are highly specific. Concerning signs in renal imaging, renal atrophy on echography is relatively rare. Renal pathology frequently reveals crescentic glomerulonephritis.

The “Clinical criteria of RPGN for early discovery of the disease,” which promotes early presentation of patients to specialists, and the “Guideline for the definite diagnosis of RPGN” are proposed as diagnostic criteria for RPGN.

Diagnostic differential criteria for diseases that manifest RPGN

Important differential diagnoses include primary vasculitis syndrome, Goodpasture syndrome, SLE, IgA vasculitis, malignancies, cryoglobulinemia, infectious diseases such as post-streptococcal acute glomerulonephritis, infectious endocarditis, and type C hepatitis infection. It is important to first exclude infectious diseases and malignancies.

## III. Epidemiology and prognosis (incidence, prevalence, and outcome)

### 1. Epidemiology

RPGN is a rare renal disease; however, the number of Japanese patients with RPGN has increased in recent years. Although the precise incidence of RPGN in Japan or worldwide is not known, a recent questionnaire survey estimated the number of new cases of RPGN in Japan at 1600–1800 per year. Based on a questionnaire survey of 1772 Japanese cases collected from 1989 to 2007, the most common clinical form of RPGN in this country is pauci-immune-type necrotizing glomerulonephritis without systemic vasculitis, and the second most common form is microscopic polyangiitis. In recent years, the age at onset has increased.

### 2. Prognosis

The survival and renal prognosis of Japanese patients with RPGN or ANCA-associated RPGN has improved in recent years. In contrast, patients with anti-GBM antibody-associated RPGN show an extremely poor prognosis. Infection has been, and continues to be, the leading cause of death in patients with RPGN.

## IV. Treatment

**1. Treatment algorithm**

Figures 1 and 2.

**Fig. 1:**
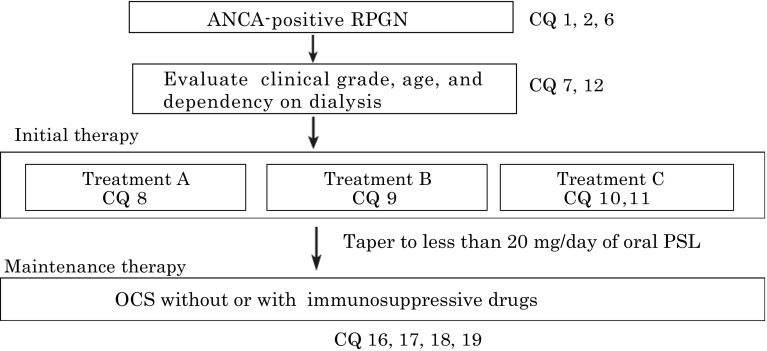
Treatment algorithm for ANCA-positive RPGN and CQs (changed from reference: the RPGN clinical practice guide 2011 by the Progressive Renal Disease Research, from the Ministry of Health, Labour and Welfare of Japan). *Asterisk* For older patients over 70 years, lower-grade treatment may be considered including the regimen without pulse methylprednisolone. *Asterisk* At the specialized hospital, higher-grade treatment may be considered under careful management irrespective of age and clinical grades. Please see other treatments (CQ 13 for Rituximab therapy, CQ 14 for Plasma exchange therapy, CQ15 for anti-coagulation and antiplatelet therapy, and CQ20 for co-trimoxazole therapy). *RPGN* rapidly progressive glomerulonephritis, *PSL* prednisolone, *OCS* oral corticosteroid

**Fig. 2:**
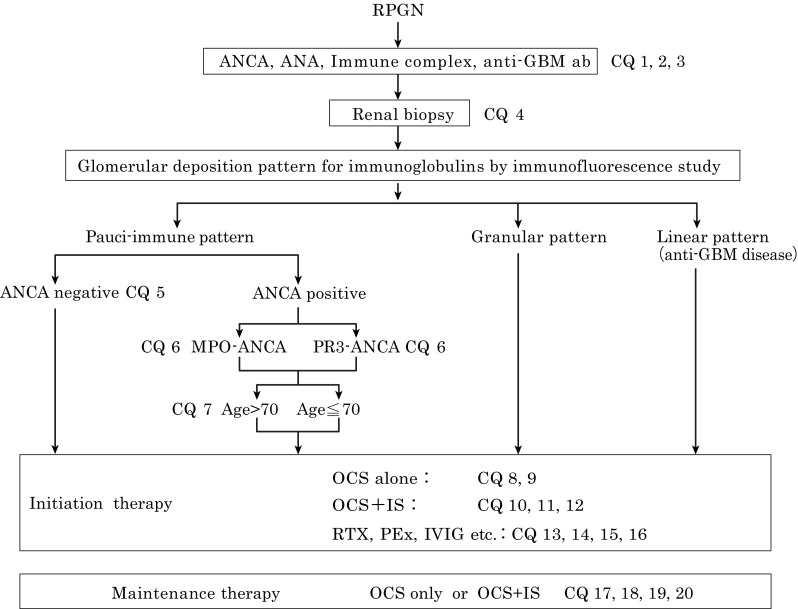
Differential diagnosis of RPGN and treatment options. The algorithm for diagnosis and treatment with corresponding CQs are shown in this figure. *OCS* oral corticosteroid, *IS* immunosuppressant, *PEx* plasma exchange, *RTX* rituximab, *IVIG* intravenous immunoglobulin

Tables 1, 2, 3, and 4.

**Table 1:** Treatment choices by clinical grades, age, and dependency on dialysis

Clinical grade on dialysis	Age ≧70 years or not on dialysis	Age <70 years and not on dialysis
I or II	A	B
III or IV	B	C

**Table 2:** The clinical grading system for predicting RPGN patient prognosis

Clinical score	Serum creatinine (mg/dL)^a^	Age (years)	Lung involvement	Serum CRP (mg/dL)^a^
0	[Cr] < 3	<60	No	<2.6
1	3 ≦ [Cr] < 6	60–69		2.6–10
2	6 ≦ [Cr]	≧70	Yes	>10
Dialysis				

Clinical grade	Total scores			
I	0–2			
II	3–5			
III	6–7			
IV	8–9			

^a^Values at the time of treatment initiation

**Table 3:** Treatment regimen

Grade	Treatment regimen
A	Oral corticosteroid alone (Prednisolone 0.6–1.0 mg/kg/day)
B	Pulse Methylprednisolone, followed by oral corticosteroid (Pulse methylprednisolone 500–1000 mg i.v. daily × 3 days, followed by oral prednisolone 0.6–0.8 mg/kg/day)
C	Pulse Methylprednisolone, followed by oral corticosteroid + oral CY (Pulse methylprednisolone 500–1000 mg i.v. daily × 3 days, followed by oral prednisolone 0.6–0.8 mg/kg/day + oral CY 25–100 mg/day)

**Table 4:** Pulsed CYC reductions for renal function and age

Age (years)	Creatinine, 1.7–3.4 mg/dL	Creatinine, 3.4–5.7 mg/dL
<60	15 mg/kg/pulse	12.5 mg/kg/pulse
60–70	12.5 mg/kg/pulse	10 mg/kg/pulse
≧70	10 mg/kg/pulse	7.5 mg/kg/pulse

Adapted from BSR and BHPR guideline for the management of adults with ANCA-associated vasculitis, 2014

**2. Clinical questions for treatment**
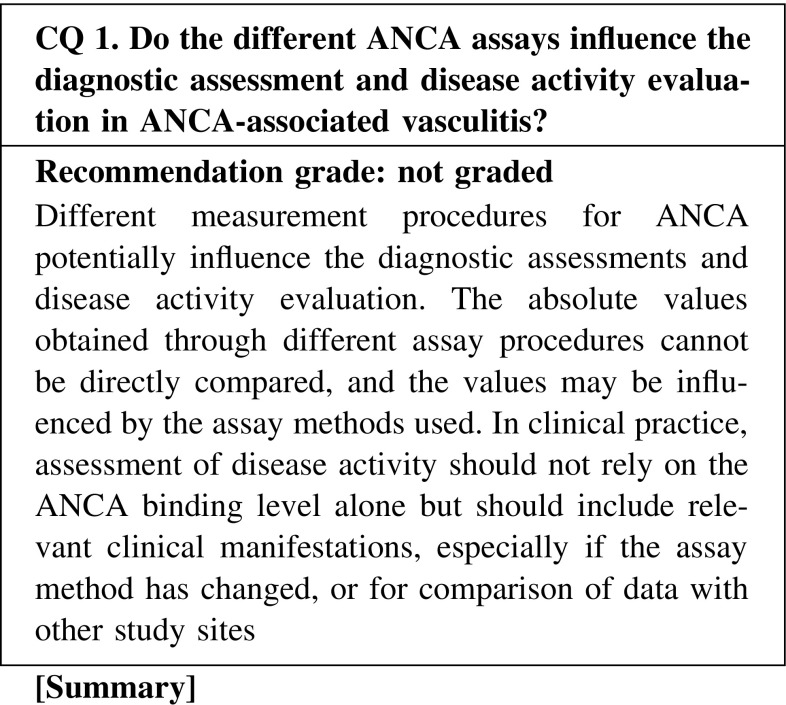


Indirect immunofluorescence (IIF) and enzyme immunoassay (EIA) have been used for ANCA testing. The labeling characteristics (cytoplasmic or perinuclear) are obtained by IIF, and identification of the specific target antigen with quantitative measurements is achieved by EIAs: enzyme-linked immunosorbent assay (ELISA), fluorescence enzyme immunoassay (FEIA), and chemiluminescent enzyme immunoassay (CLEIA). The different procedures for the measurement of ANCA affect the diagnostic assessments and disease activity evaluation. The absolute values obtained through different assays cannot be directly compared, and multicenter clinical/epidemiological studies need to consider the differences in assay methods when comparing data. It should also be noted that assessment of disease activity should not rely on the ANCA binding level alone, but should be evaluated together with clinical manifestations, especially when using data obtained at different times with different methods. The absence of a positive test does not rule out a diagnosis. Duplicated serial measurements or measurements with both IIF and EIA are recommended for making decisions concerning positivity and negativity (Fig. 2). 
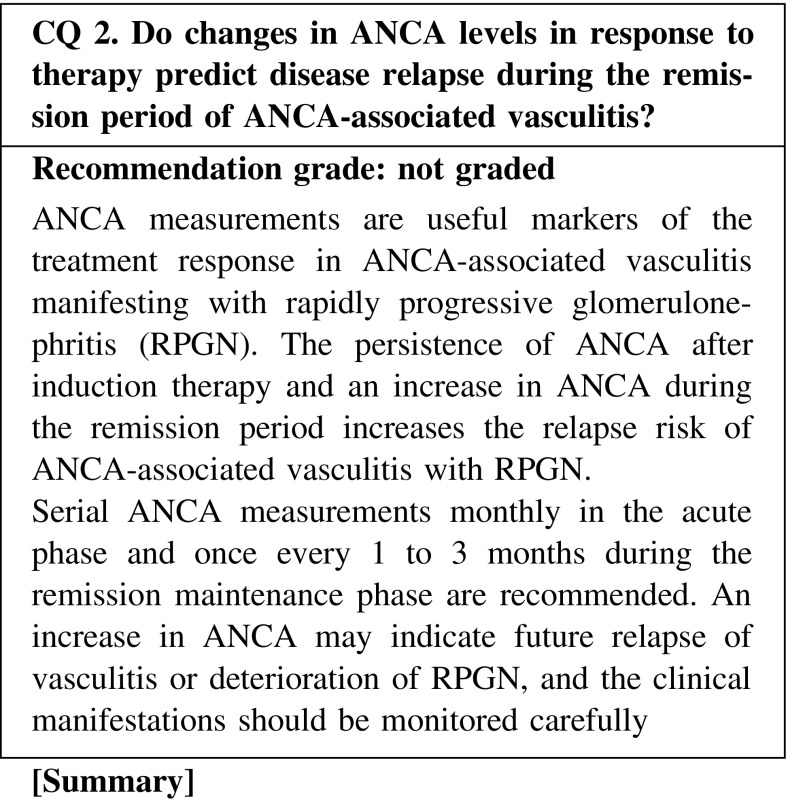


Remission is defined as the absence of disease activity after a course of induction treatment for ANCA-associated vasculitis. The remission maintenance phase is defined as the period of sustained absence of disease activity. Relapse is a new or recurrent disease activity that occurs after remission has been initially induced. There are no definitions for “remission” and “relapse” in RPGN.

The ANCA binding level usually decreases in response to the treatment; thus, it is a useful marker that reflects disease activity. Persistent ANCA may occur in some cases. Treatment should not be tapered solely based on the ANCA level, and a comprehensive evaluation with careful observation of clinical symptoms and other physical/laboratory manifestations is required.

Persistence of ANCA positivity after induction therapy or an increase in ANCA during the remission phase increases the risk of relapse in ANCA-associated vasculitis. It is recommended to check the ANCA level once every 1–3 months during the remission maintenance phase. There is a lack of evidence to support changing of treatment to prevent disease relapse based on the reappearance of ANCA or an increase in ANCA binding level during the remission maintenance phase. An increase in ANCA indicates an increase in relapse risk, and clinical manifestations should be monitored carefully. Treatment should not be escalated solely because of an increase in ANCA.
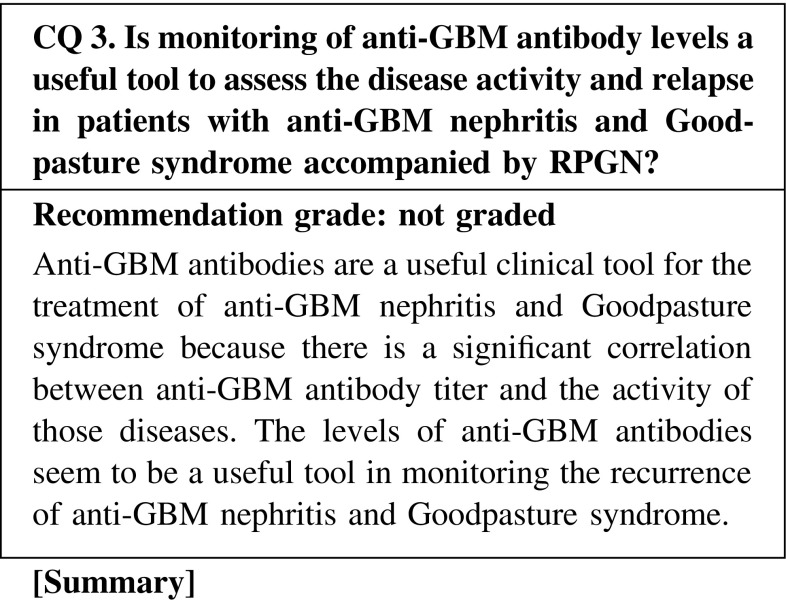


Anti-GBM disease, also known as Goodpasture disease, is an autoimmune disorder characterized by rapidly progressive glomerulonephritis (RPGN) and a high risk for alveolar hemorrhage. Anti-GBM antibodies have been proven to be pathogenic in disease initiation. The target GBM antigen for circulating antibodies was subsequently identified as the non-collagenous-1 (NC1) domain of the α3 chain of collagen IV, whereas further studies revealed that collagen IV is a family of six α-chains (α1 through α6). Two major immunodominant regions, EA and EB, have been mapped to residues 17–31 and 127–141 of α3(IV)NC1. Antibodies against linear epitopes on the Goodpasture autoantigen could be detected in human anti-GBM disease and were associated with kidney injury. Another study defines them as conformational epitopes that are sequestrated in the quaternary structure of GBM dependent on a critical sulfilimine bond.

No high-level evidence exists from published clinical trials on the association between anti-GBM antibody levels and disease activity, although many experiment-based studies are well established. According to a retrospective study, high antibody titers at diagnosis seemed to be associated with poor renal and patient survival. Therefore, treatment with plasmapheresis in combination with immunosuppression is recommended to remove the antibodies. In patients with a recurrence of anti-GBM disease, the anti-GBM level is useful in the diagnosis and in deciding the therapy.
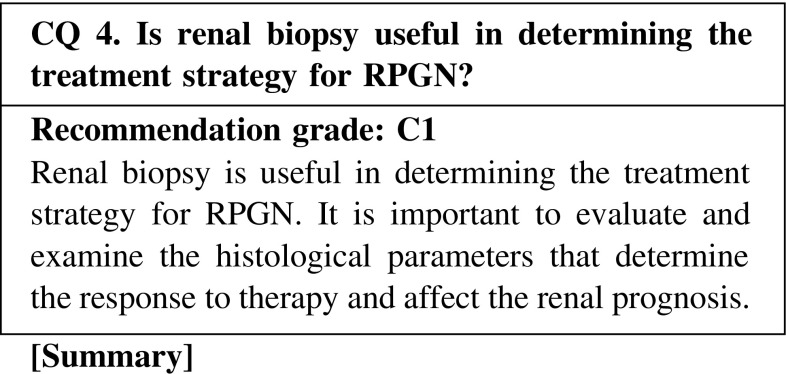


Evidence for the necessity to perform treatment, along with the presence of adverse effects, can be obtained through renal biopsy when the findings show reversible lesions. Excess immunosuppression can be prevented if the findings show irreversible changes. Thus, renal biopsy is useful in determining the treatment strategy for RPGN. On the other hand, treatment should be prioritized in patients who are positive for ANCA or anti-GBM antibody and are at high risk of complications with renal biopsy. In most papers, the renal prognosticator of ANCA-associated nephritis has been reported to be the percentage of normal glomeruli. A scoring system for glomerular, tubulointerstitial, and vascular lesions of ANCA-associated vasculitis was proposed in Japan in 2008. European Vasculitis Society (EUVAS) proposed the new classification stratified only based on glomerular lesions. In anti-GBM glomerulonephritis, most papers report the percentage of crescents to be the renal prognosticator.
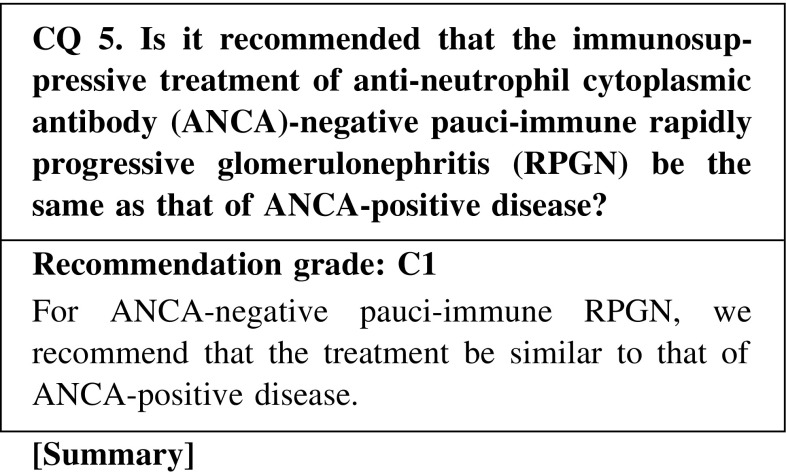


Reports from Japan and other countries showed that some patients with pauci-immune RPGN lacked ANCA. Some showed that there were no differences between patients with ANCA and those without ANCA; however, other studies reported the opposite. Because treatment of ANCA-negative pauci-immune RPGN has not been discussed in detail, we recommend that the treatment be similar to that of ANCA-positive disease.
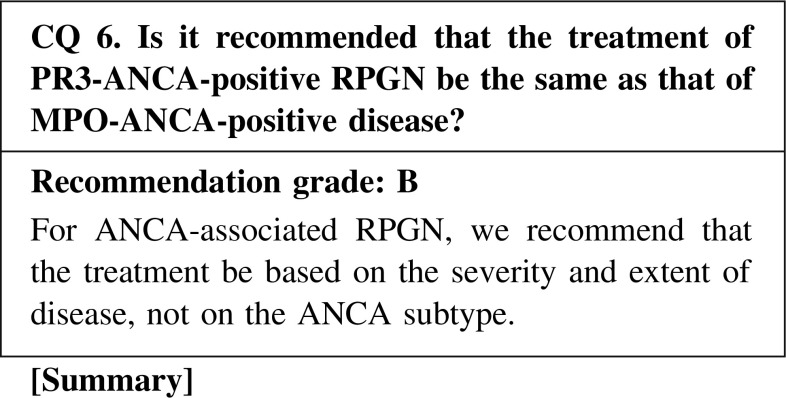


PR3-ANCA-positive RPGN is more common in Europe and the United States, whereas MPO-ANCA-positive RPGN is more common in Japan. Therefore, the treatment in Europe and the United States, which focuses on PR3-ANCA-positive RPGN, should not be directly adopted in Japan. However, the recent treatments introduced in Europe and the United States as well as in Japan are based on the severity and extent of disease, and not on the ANCA subtype. In fact, in Europe and the United States as well as in Japan, no differences in renal outcome and survival were observed between ANCA subtypes. However, special care should be taken to prevent relapse of PR3-ANCA-positive RPGN.
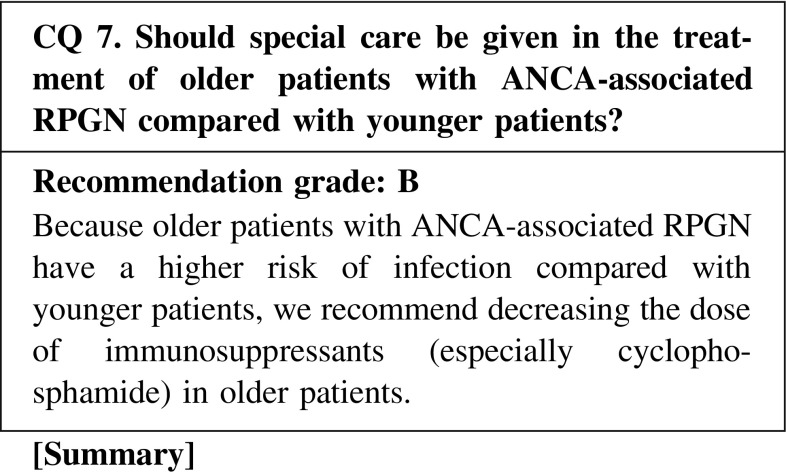


Patients with RPGN in Japan are older compared with those in Europe and the United States. Recently, Japanese patients with RPGN have shown better survival. Therefore, we recommend preventing infection due to over-immunosuppression in patients older than 70 years old, although they may have a higher risk of relapse. Infection is the most common and severe complication of ANCA-associated vasculitis in Europe and the United States, as well as in Japan. It is recommended that older patients, especially those with poor renal function, should be given reduced cyclophosphamide dose according to their age. Furthermore, steroids could cause serious adverse events such as diabetes mellitus, bone fractures, and cerebrovascular accidents, as well as infection. Careful attention should be given to the dose given to older patients to prevent the high incidence of serious adverse events with the use of several drugs.
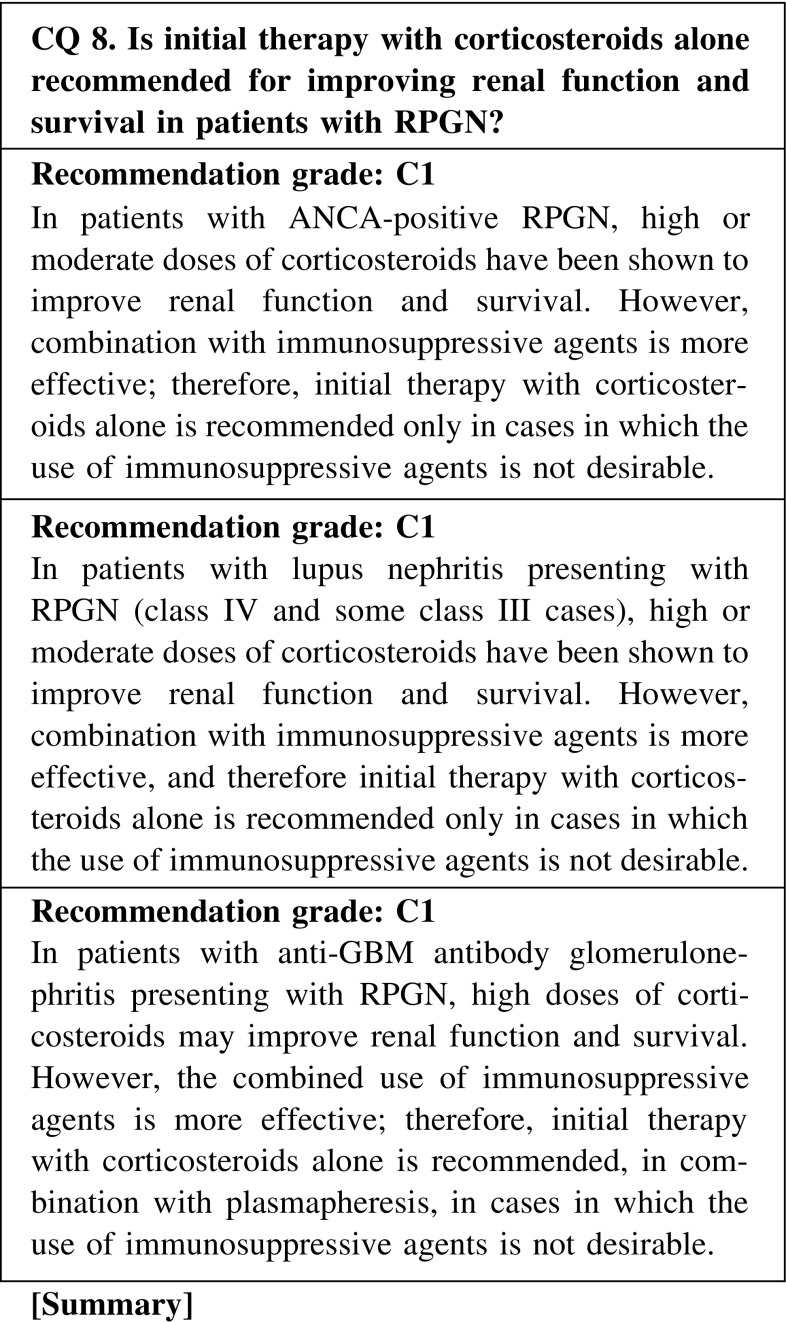


In patients with ANCA-positive RPGN, the combined use of corticosteroids and immunosuppressive agents is currently recommended as the standard therapy, and there are no randomized controlled trials (RCTs) that compared treatment with and without corticosteroids. Therefore, initial therapy with corticosteroids alone is indicated only in cases in which aggressive treatment is required but the use of immunosuppressive agents is not desirable, such as in patients in whom systemic infection is present or cannot be ruled out, thus conferring increased risk by addition of immunosuppressive agents, dialysis-dependent patients, elderly patients (particularly those older than 70 years), and those in whom immunosuppressive agents are contraindicated because of leukopenia and liver dysfunction.

In patients with lupus nephritis presenting with RPGN (class IV and some class III cases), the combined use of corticosteroids and immunosuppressive agents is the current standard therapy. Therefore, initial therapy with corticosteroids alone is indicated only in cases in which aggressive treatment is required to prevent the progression of renal disease or to improve severe systemic complications in other vital organs, including the lung and the central nervous system, but in which the use of immunosuppressive agents is not desirable.

The prognosis of anti-GBM antibody disease is poor without treatment, with the worst patient survival in the presence of pulmonary hemorrhage. In patients with anti-GBM antibody glomerulonephritis presenting with RPGN, the combined use of corticosteroids and immunosuppressive agents, in addition to plasmapheresis, is suggested as the standard treatment. Therefore, initial therapy with corticosteroids alone is recommended, usually combined with plasmapheresis, in cases in which the use of immunosuppressive agents is not desirable because of adverse effects.
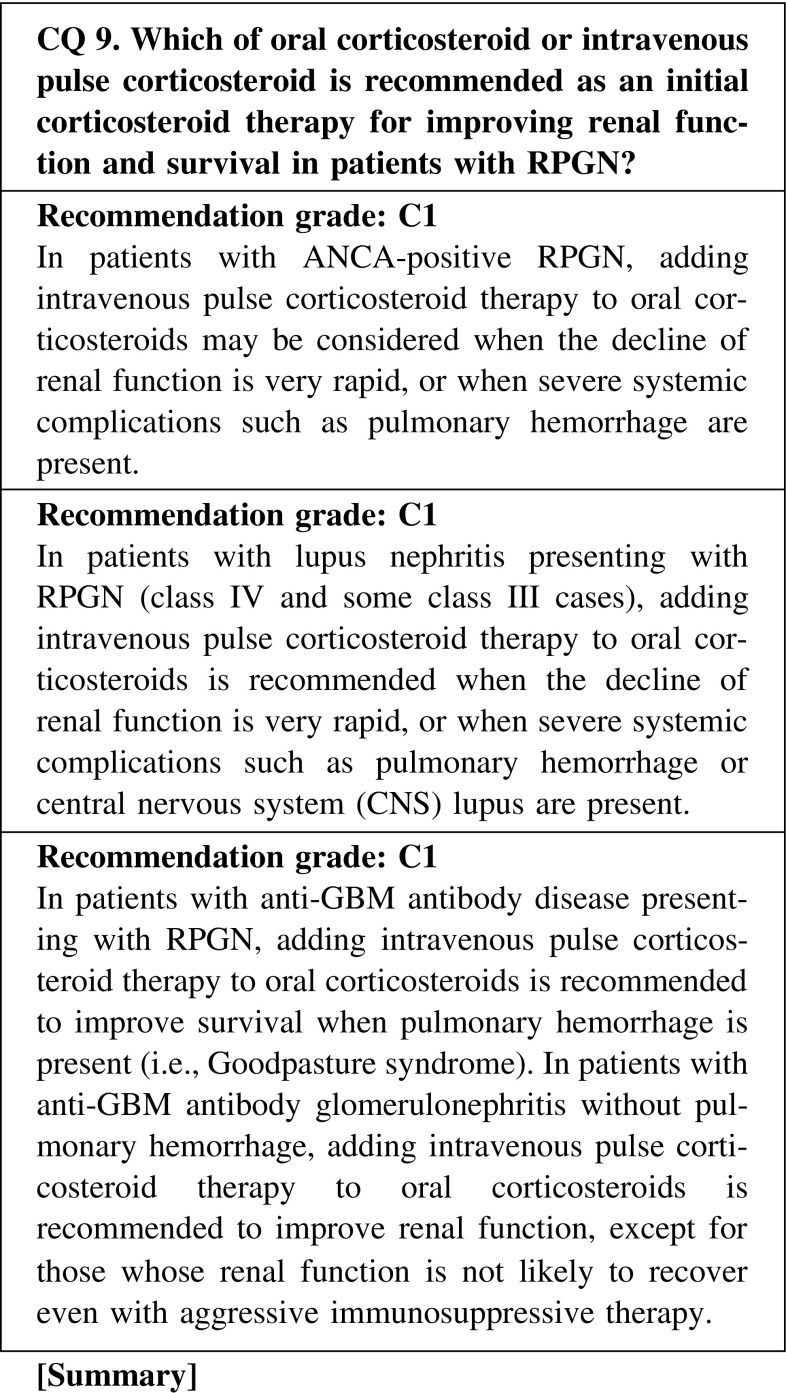


In ANCA-positive glomerulonephritis, lupus nephritis (class IV and some class III cases), or anti-GBM antibody glomerulonephritis presenting as RPGN, there are no RCTs that have compared the effect on renal survival or patient survival between oral corticosteroids and intravenous pulse corticosteroid therapy. However, this is considered to confer rapid, strong anti-inflammatory and immunosuppressive effects in patients with high disease activities such as

- ANCA-positive glomerulonephritis, in which the decline of renal function is very rapid or is associated with severe systemic complications, including pulmonary hemorrhage
- Lupus nephritis presenting with RPGN (class IV and some class III cases), in which the decline of renal function is very rapid or is associated with severe systemic complications, including pulmonary hemorrhage and CNS lupus
- Anti-GBM antibody glomerulonephritis presenting with RPGN but without pulmonary hemorrhage, except for those whose renal function is not likely to recover despite aggressive therapy, or almost all cases of Goodpasture syndrome that is complicated by pulmonary hemorrhage

The standard protocol in pulse corticosteroid therapy is intravenous administration of 500 mg to 1 g of methylprednisolone for three consecutive days, followed by 0.6–0.8 mg/kg body weight of oral prednisolone.
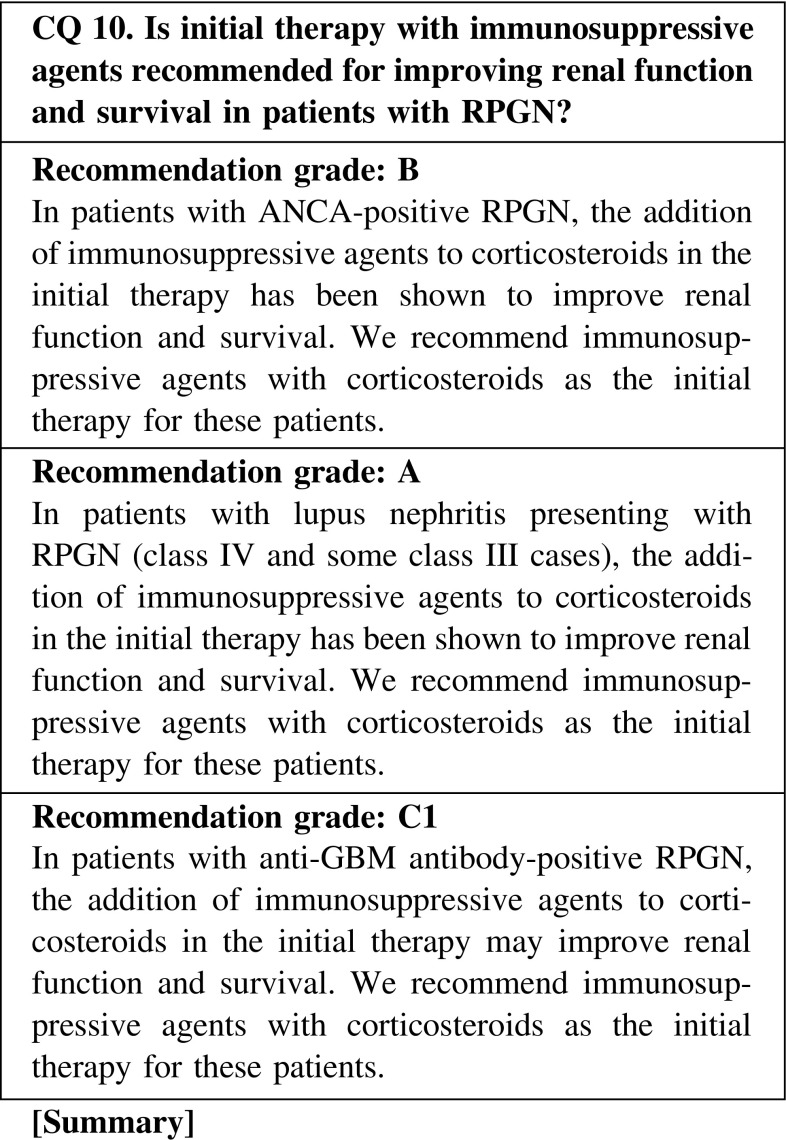

1. ANCA-positive RPGN

Treatment with corticosteroids and cyclophosphamide has improved the outcome of patients with ANCA-positive RPGN. We recommend daily oral cyclophosphamide (25–100 mg/day) or intravenous pulses of cyclophosphamide (250–750 mg/m^2^/month) with corticosteroids as the initial therapy, considering the clinical grade, patient age, and dialysis requirement.

(2) Lupus nephritis presenting with RPGN

We recommend immunosuppressive agents (cyclophosphamide or mycophenolate mofetil) with corticosteroids as the initial therapy for patients with diffuse proliferative lupus nephritis.

(3) Anti-GBM antibody-positive RPGN

Patient survival and kidney survival in anti-GBM antibody-positive RPGN are poor. The clinical guideline in Japan recommends immunosuppressive therapy (corticosteroids and cyclophosphamide) plus plasmapheresis. We recommend cyclophosphamide (1–2 mg/kg/day) for patients with refractory GN. However, it is necessary to reduce the dose of cyclophosphamide in patients with advanced renal dysfunction.
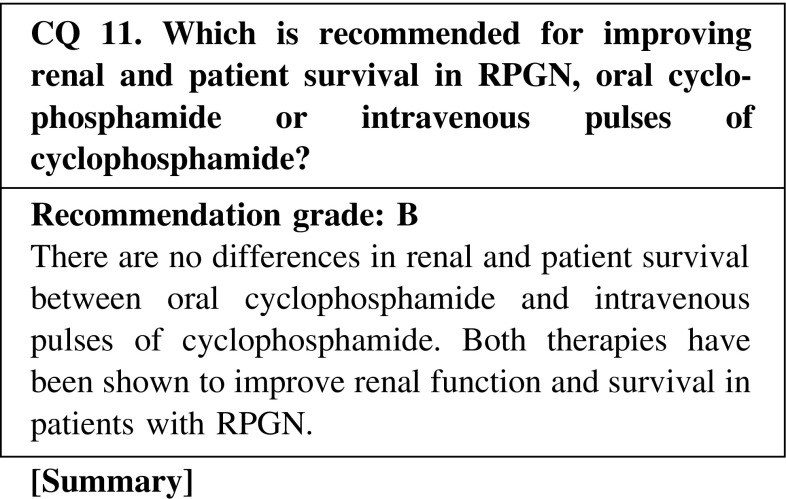


The clinical guideline in Japan recommends immunosuppressive agents with corticosteroids as the initial therapy, considering the clinical grade, patient age, and dialysis requirement. The guideline recommends daily oral cyclophosphamide (25–100 mg/day) or intravenous pulses of cyclophosphamide (250–750 mg/m^2^/day/month) in patients with clinical grade I and II in whom the effects of corticosteroids are not enough, and in patients with clinical grade III and IV who are younger than 70 years. There are no differences in renal and patient survival between oral cyclophosphamide and intravenous pulses of cyclophosphamide, although treatment with intravenous pulses of cyclophosphamide has reduced the rate of relapse and adverse events.
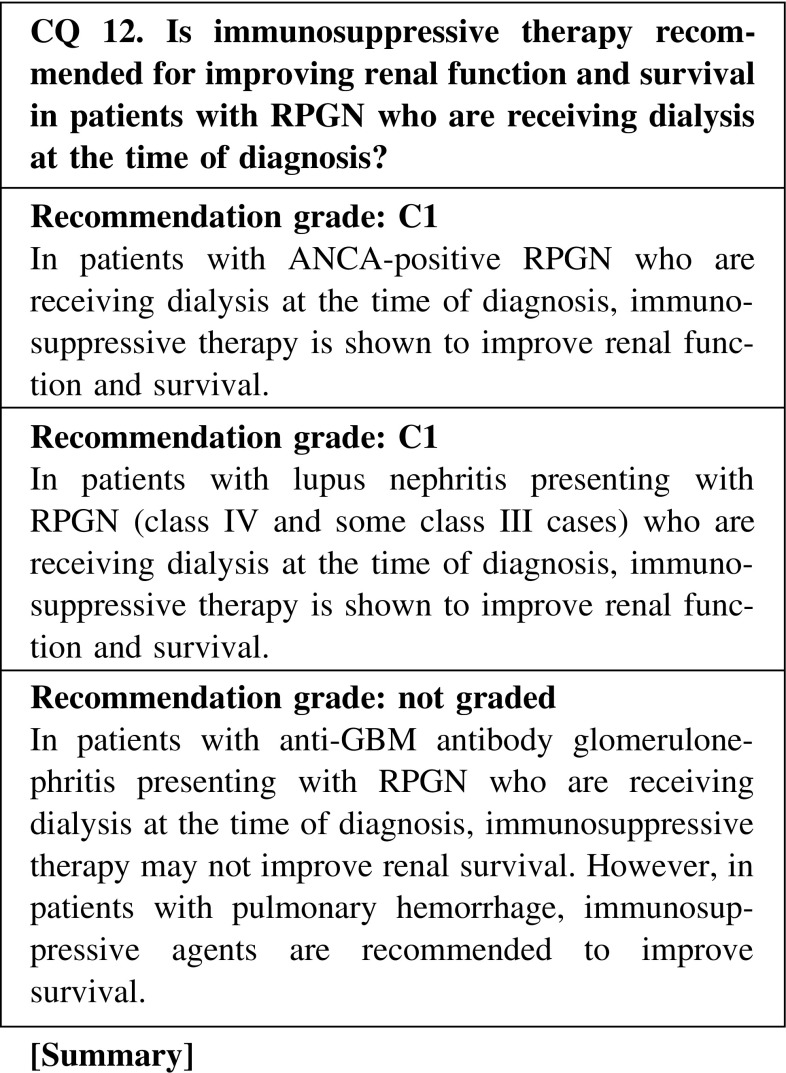


In patients with granulomatosis with polyangiitis (GPA) or microscopic polyangiitis (MPA) who have severe active renal disease, the addition of plasma exchange to cyclophosphamide and glucocorticoid therapy is currently recommended by the European league against rheumatism (EULAR) guideline. Even in patients with dialysis-dependent ANCA-associated vasculitis, the chance of renal recovery is high when they have a high percentage of normal glomeruli. However, as therapy-related deaths usually occur in older patients and in those with poor general condition, carefully decisions for safer treatment regimens are warranted.

In patients with lupus nephritis presenting with RPGN (class IV and some class III cases), the combined use of corticosteroids and immunosuppressive agents such as intravenous cyclophosphamide or mycophenolate mofetil is the current standard therapy by American College of Rheumatology (ACR) guideline. Liang reported that 59.3 % patients with lupus nephritis with recent-onset renal failure recovered their renal function after 6 months of follow-up, whereas 11.1 % had died. As the chronic component of renal function loss is often irreversible with immunosuppressive therapy, renal echogram and renal biopsy should be performed to determine whether the renal failure is reversible.

In patients with anti-GBM antibody glomerulonephritis presenting with RPGN who are receiving dialysis at the time of diagnosis, immunosuppressive therapy may not improve renal survival. However, in patients with pulmonary hemorrhage, immunosuppressive agents are recommended to improve survival.
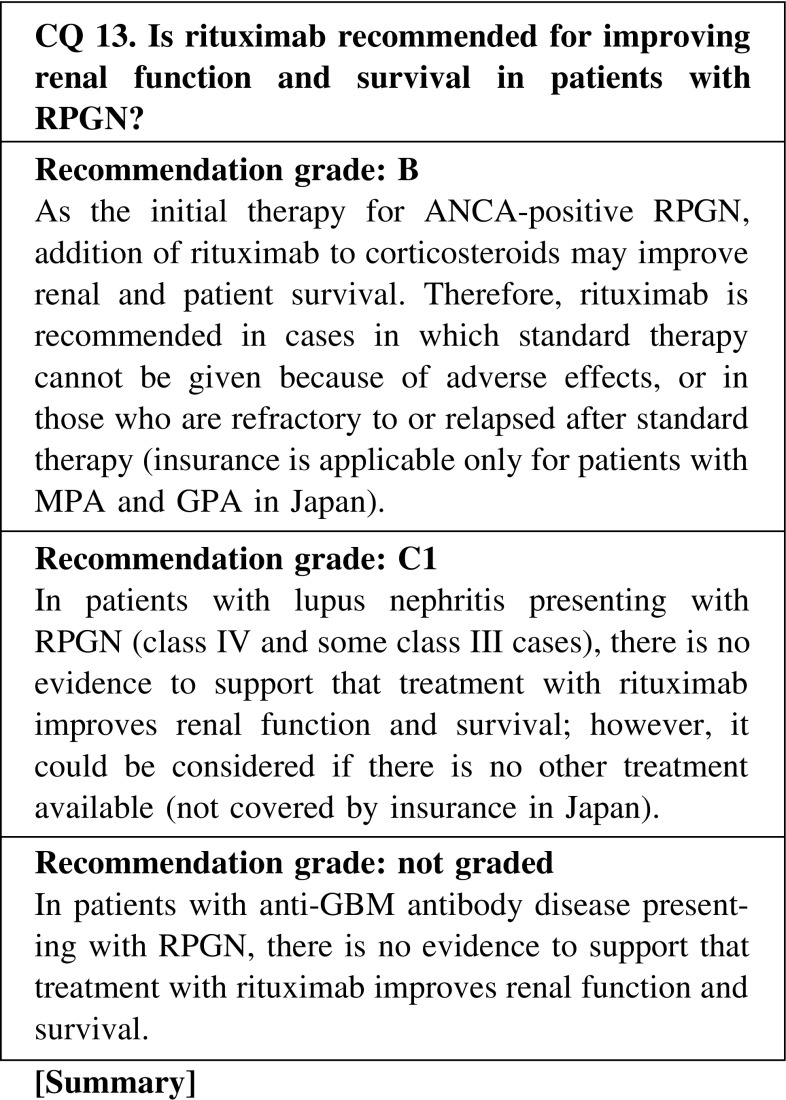


B-cell-targeted therapy has recently been introduced for patients with ANCA-associated vasculitis, considering that production of ANCA may be involved in the pathogenesis of this disease. Based on the promising results of two recent RCTs, rituximab has just become available in Japan, as well as in the United States and Europe, but only for cases in which standard therapy cannot be given because of adverse effects or in patients who are refractory to or relapsed after standard therapy. However, the patient profiles of renal-limited ANCA-positive or MPO-ANCA-associated RPGN, which is more common in Japan, were not described in those trials. Moreover, there is a substantial risk of infection, as well as concerns about long-term safety concerning the incident risk of malignancy and leukoencephalopathy. Thus, it is necessary to perform screening tests to detect infection and to take preventive measures before starting rituximab. Furthermore, careful follow-up to detect the occurrence of infection and other adverse events is mandatory after the administration of rituximab.

B-cell-targeted therapy has been used for patients with SLE to suppress antibody production and immune complex formation. However, in lupus nephritis presenting with RPGN (class IV and some class III cases), there have been no RCTs that demonstrate the superiority of B-cell-targeted therapy over standard immunosuppressive therapy. Therefore, the use of rituximab may be considered only if standard therapy cannot be given because of adverse effects, or in patients who are refractory to or relapsed after standard therapy.

In patients with anti-GBM antibody disease with or without pulmonary hemorrhage, a treatment regimen including rituximab has been attempted for suppressing the production of anti-GBM antibody, and evidence is accumulating that suggests its effectiveness. However, rituximab is usually given concomitant with other drugs such as corticosteroids, cyclophosphamide, and plasmapheresis; thus, at present, there is no sufficient evidence that rituximab itself is actually effective.
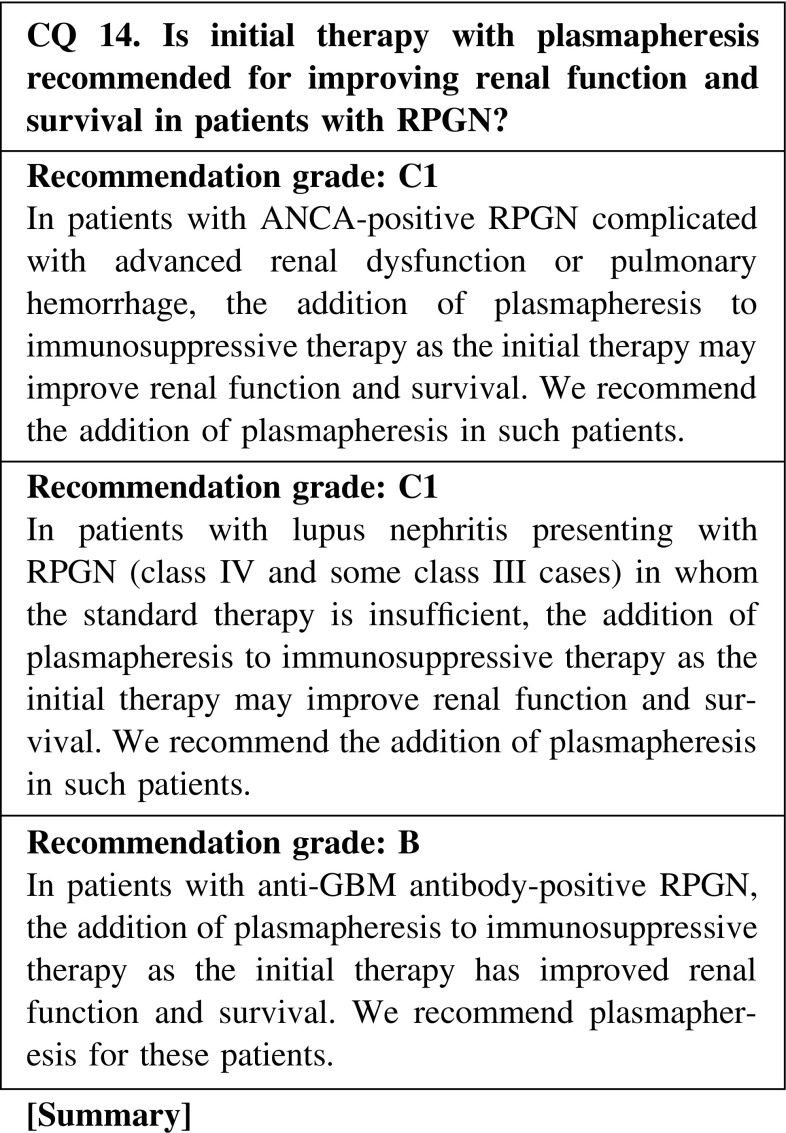

1. ANCA-positive RPGN

ANCA is thought to be involved in the clinical conditions of ANCA-associated vasculitis and RPGN. The removal of ANCA may therefore result in controlling disease activity and preventing organ damage. The addition of plasmapheresis to the initial therapy with corticosteroids and cyclophosphamide is indicated for patients presenting with advanced kidney failure (serum creatinine, >5.8 mg/dL) or those with diffuse alveolar hemorrhage.

(2) Lupus nephritis presenting with RPGN

The addition of plasmapheresis to the initial therapy is indicated for patients in whom the standard therapy (corticosteroids and immunosuppressive agents) is insufficient.

(3) Anti-GBM antibody-positive RPGN

We recommend the addition of plasmapheresis for improving renal function and survival in patients with anti-GBM antibody-positive RPGN. On the other hand, in patients with advanced kidney failure or a requirement for dialysis, there is rare evidence that the addition of plasmapheresis improves renal function and survival.

(4) Medical care insurance

Patients with SLE presenting with RPGN have insurance coverage for plasmapheresis. However, plasmapheresis for patients with ANCA-positive RPGN and anti-GBM antibody-positive RPGN is not covered by the medical care insurance in Japan.
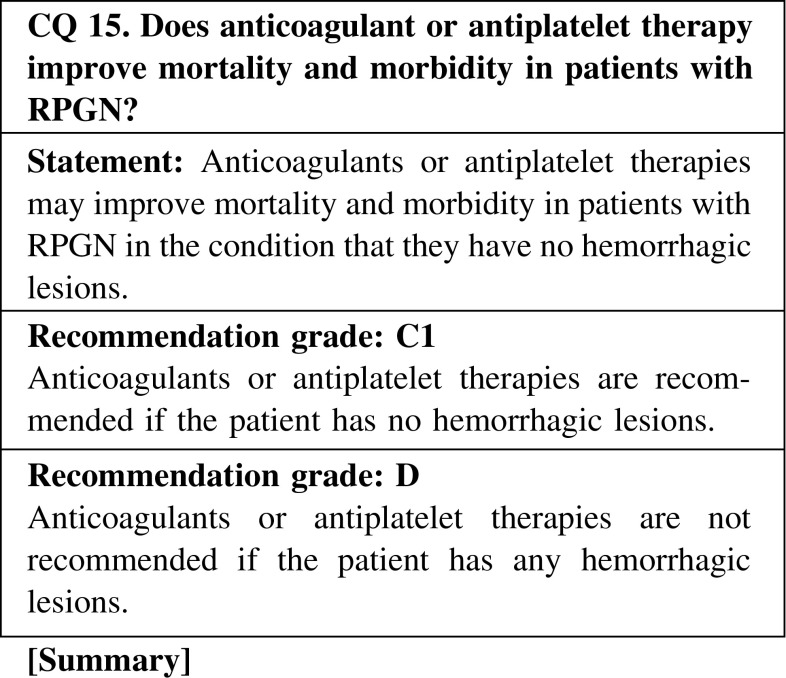


The efficacy of anticoagulant or antiplatelet therapy in improving mortality and morbidity in the treatment of rapidly progressive glomerulonephritis has not been established by solid evidences. However, anticoagulants such as heparin and warfarin or antiplatelet therapies with aspirin and eicosapentaenoic acid were reported to be helpful in the treatment of ANCA-associated vasculitis in some cases. In fact, these agents are sometimes used to prevent thrombosis-associated cardiovascular events, especially in patients treated with steroids. On the other hand, as pulmonary hemorrhage and/or gastrointestinal bleeding can occur as complications in ANCA-associated vasculitis, careful attention should be given to treatment with anticoagulants and antiplatelet drugs.
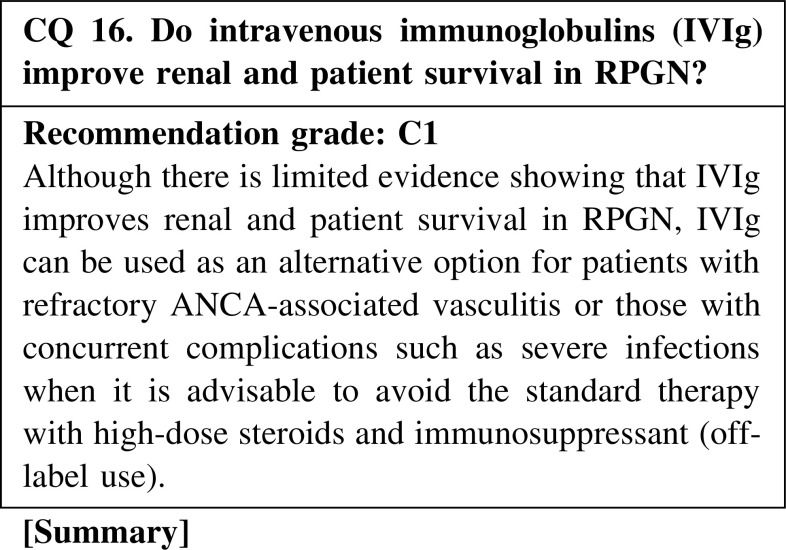


IVIg can be used as an alternative option for patients with refractory ANCA-associated vasculitis or those with concurrent complications such as severe infections when the optimal standard therapy with high-dose steroids and immunosuppressant is not recommended (off-label use). Sulfonated immunoglobulin has been used according to label directions for refractory peripheral neuropathy caused by eosinophilic granulomatosis with polyangiitis/Churg–Strauss syndrome since 2010 in Japan, and it has been reported to improve polyneuropathy and cardiac function, as well as to have a steroid sparing effect. In addition, a clinical trial to evaluate the efficacy for MPA with peripheral neuropathy has been initiated. Thus, IVIg might improve renal and patient survival in RPGN, although evidence is lacking thus far and there is a need for further evaluation in clinical trials.
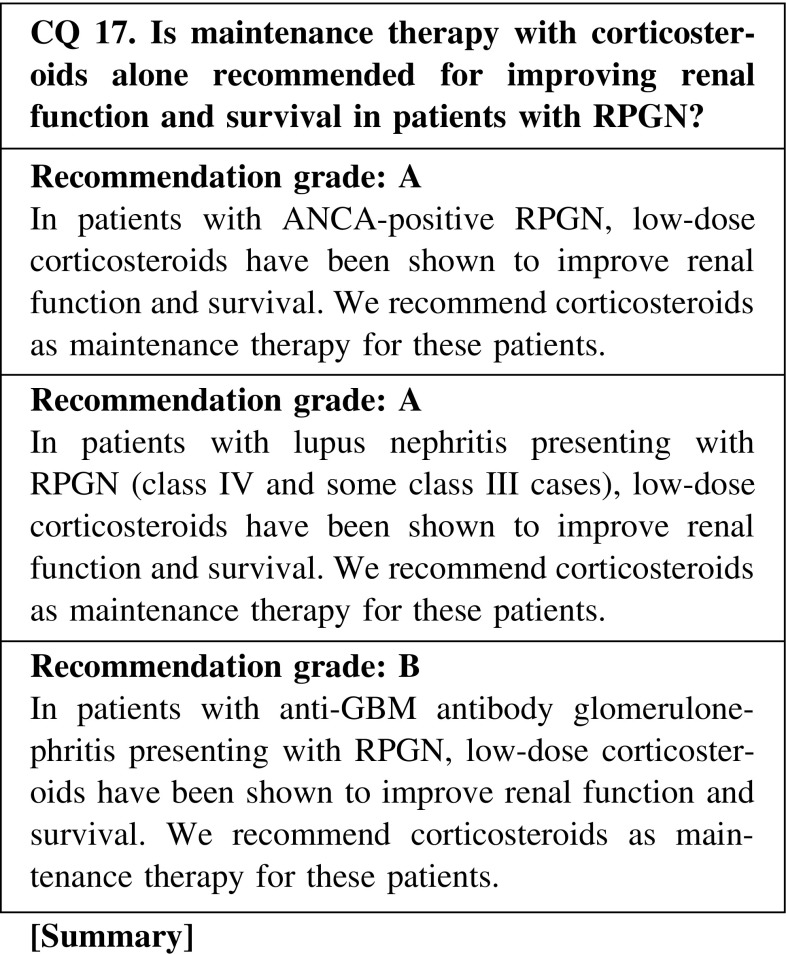


Maintenance immunosuppressive therapy for RPGN may prevent relapse, although it may also increase the risk of opportunistic infection. Therefore, it is necessary to consider the total duration of treatment and the dose of corticosteroids in maintenance therapy to prevent relapse and opportunistic infection.

1. ANCA-positive RPGN

We recommend a corticosteroid dose of <10 mg/day orally as maintenance therapy, and suggest continuing administration for 12–18 months in patients who remain in complete remission. A study reported that a reduction rate >0.8 mg/month was associated with a higher relapse rate. Shortening the treatment period should be considered in aged or dialysis-dependent patients.

(2) Lupus nephritis presenting with RPGN

We recommend continuing low-dose corticosteroids (5–7.5 mg/day) orally as maintenance therapy in patients with lupus nephritis presenting with RPGN.

(3) Anti-GBM antibody-positive RPGN

There is rare evidence suggesting the efficacy of low-dose corticosteroids in patients with anti-GBM antibody-positive RPGN. We suggest continuing corticosteroids for 6–12 months as maintenance therapy.
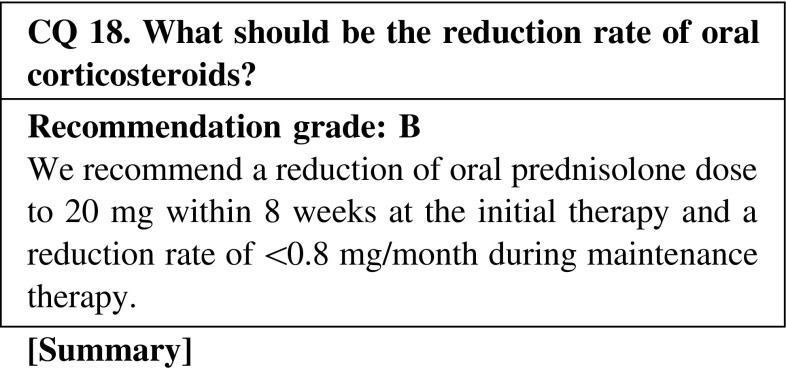


We recommend a reduction of the oral prednisolone dose to 20 mg within 8 weeks at the initial therapy to prevent opportunistic infection. However, a too early decrease in the amount of steroid was reported to be a risk factor for relapse, and the recommended reduction rate of the oral prednisolone dose during maintenance therapy is <0.8 mg/month.
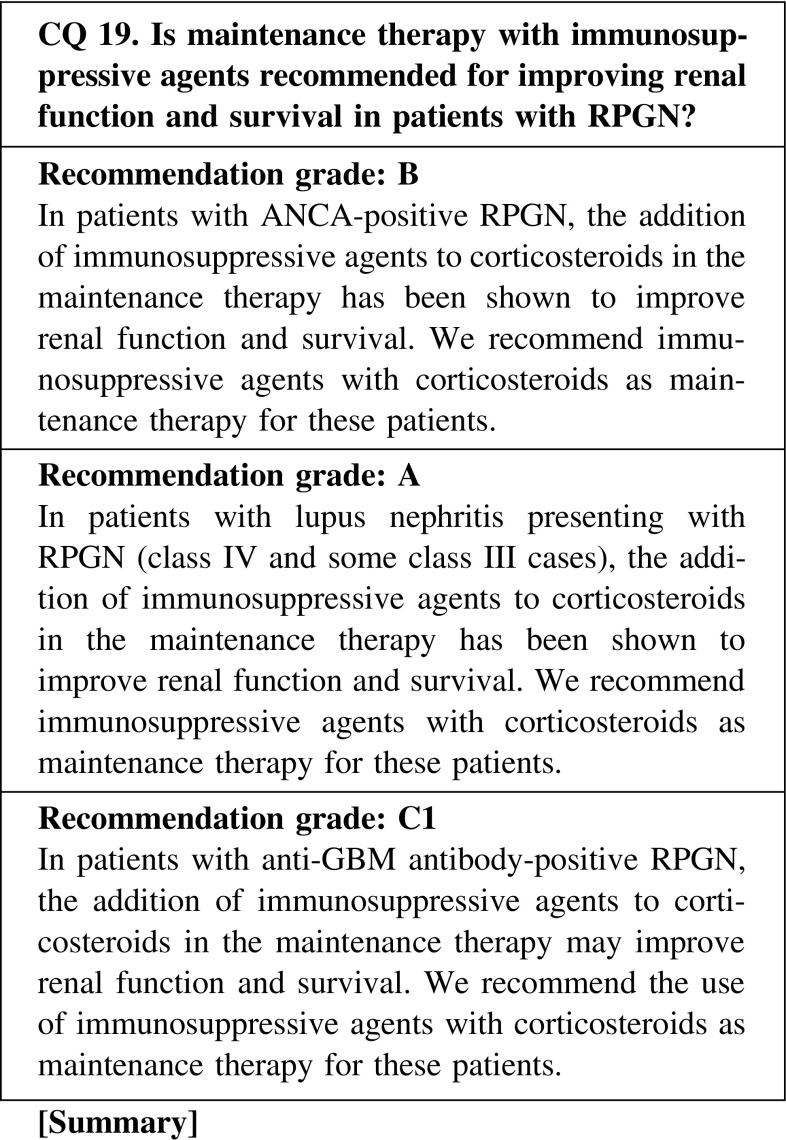


Maintenance immunosuppressive therapy for patients with RPGN may prevent relapse; however, it may also increase the risk of opportunistic infection. Therefore, it is necessary to consider immunosuppressive agents as maintenance therapy to prevent relapse and opportunistic infection. We recommend treatment with azathioprine or mizoribine in patients with ANCA-positive RPGN, and mycophenolate mofetil or azathioprine in patients with lupus nephritis presenting with RPGN as maintenance therapy to prevent relapse.

1. ANCA-positive RPGN

The effectiveness of cyclophosphamide along with azathioprine, mizoribine, mycophenolate mofetil, and methotrexate as immunosuppressive agents in patients with ANCA-associated vasculitis has been reported. We recommend either azathioprine or mizoribine in combination with corticosteroids as maintenance therapy in patients with ANCA-positive RPGN, to prevent relapse.

(2) Lupus nephritis presenting with RPGN

The effectiveness of azathioprine and mycophenolate mofetil as immunosuppressive agents in patients with lupus nephritis has been reported. We recommend either azathioprine or mycophenolate mofetil in combination with corticosteroids as maintenance therapy in patients with lupus nephritis presenting with RPGN, to prevent relapse.

(3) Anti-GBM antibody-positive RPGN

There is rare evidence in patients with anti-GBM antibody-positive RPGN. We suggest continuing corticosteroids and immunosuppressive agents (azathioprine, etc.) for 6–12 months as maintenance therapy.
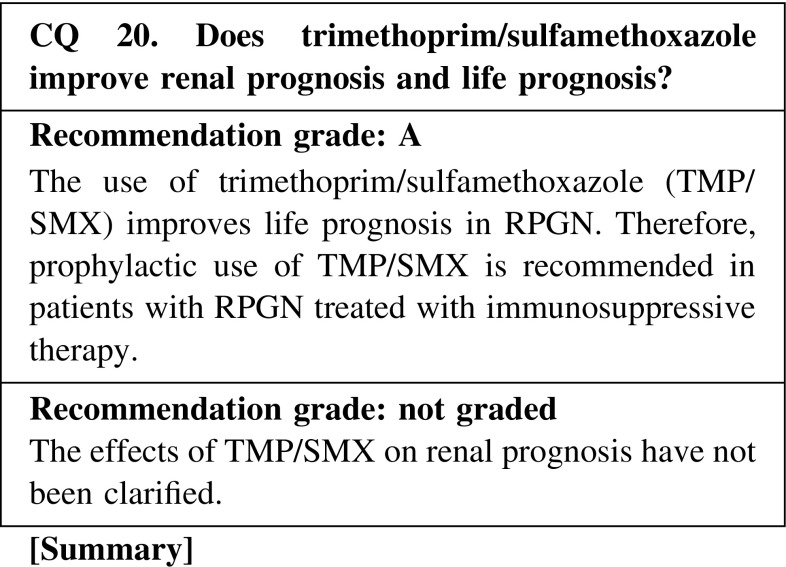


The rate of pneumocystis pneumonia (PCP) without the prophylactic use of TMP/SMX has been reported to be 4.0 or 17.6 % in Japan. In other countries, the rate of PCP has been reported to be 1, 6, or 20 %. The doses of corticosteroids and cyclophosphamide used may be related with the incidence. The mortality rate after the onset of PCP has been reported to be 9–60 %. When TMP/SMX was administered, a 91 % reduction of PCP incidence rate was observed and PCP-related mortality was significantly reduced according to a systematic review and meta-analysis of randomized controlled trials of PCP prophylaxis for immunocompromised non-HIV-infected patients.

### **Acknowledgments**

All authors are advisory committee members of Clinical Guidelines for Rapidly Progressive Glomerulonephritis 2014. Committee chairman: Yoshihiro Arimura. Committee member: Eri Muso, Shoichi Fujimoto, Midori Hasegawa, Shinya Kaname, Joichi Usui, Toshiko Ihara, Masaki Kobayashi, Itabashi Mitsuyo, Kiyoki Kitagawa, Junichi Hirahashi. Chief Chairman of the Clinical Practice Guidelines for Progressive Kidney Diseases: Kenjiro Kimura (Cooperative Medical Society). Leader of the Research for Progressive Kidney Diseases of the Ministry of Health, Labour and Welfare: Seiichi Matsuo (Nagoya University). Cooperative Medical Society: The Japanese Association for Infectious Diseases, The Japan College of Rheumatology.

**Bibliography**

I. Disease entity·definition (pathogenesis·pathophysiology)

1. Jennette JC, et al. 2012 revised International Chapel Hill consensus conference nomenclature of vasculitides. Arthritis Rheum. 2013;65:1–11.
2. Pedchenko V, et al. Molecular architecture of the Goodpasture autoantigen in anti-GBM nephritis. N Engl J Med. 2010;363:343–54.
3. Levy JB, et al. Clinical features and outcome of patients with both ANCA and anti-GBM antibodies. Kidney Int. 2004;66:1535–40.
4. Rutgers A, et al. Coexistence of anti-glomerular basement membrane antibodies and myeloperoxidase-ANCAs in crescentic glomerulonephritis. Am J Kidney Dis. 2005;46:253—62.
5. Yang R, et al. Antigen and epitope specificity of anti-glomerular basement membrane antibodies in patients with Goodpasture disease with or without anti-neutrophil cytoplasmic antibodies. J Am Soc Nephrol. 2007;18:1338–43.
6. Olson SW, et al. Asymptomatic autoantibodies associate with future anti—glomerular basement membrane disease. J Am Soc Nephrol. 2011;22:1946–52.
7. Belmont HM, et al. Pathology and pathogenesis of vascular injury in systemic lupus erythematosus. Interactions of inflammatory cells and activated endothelium. Arthritis Rheum. 1996;39:9–22.

II. Diagnosis (symptoms and signs)

1. Matsuo S, et al. Guidelines for the treatment of rapidly progressive glomerulonephritis, second version. Nihon Jinzo Gakkai Shi. 2011;53:509–55
2. Guidelines for the management of rapidly progressive glomerulonephritis. Nihon Jinzo Gakkai Shi. 2002;44:55–82.
3. Shigematsu H, et al. Glomerulointerstitial events in rapidly progressive nephritic syndrome, with special reference to histologic grade and stage on the renal lesions. Clin Exp Nephrol. 1998;2:330–8.
4. Joh K, et al. Renal pathology of ANCA—related vasculitis: proposal for standardization of pathological diagnosis in Japan. Clin Exp Nephrol. 2008;12:277–91.
5. Berden AE, et al. Histopathologic classification of ANCA-associated glomerulonephritis. J Am Soc Nephrol. 2010;21:1628–36.
6. Chang DY, et al. Re-evaluation of the histopathologic classification of ANCA—associated glomerulonephritis: a study of 121 patients in a single center. Nephrol Dial Transplant. 2012;27:2343–9.
7. Muso E, et al. Evaluation of the newly proposed simplified histological classification in Japanese cohorts of MPO-ANCA—associated glomerulonephritis in comparison with other Asian and European cohorts. Clin Exp Nephrol. 2013;17:659–62.
8. Kussmaul A, et al. Ubereinenichtbisherbeschriebeneeigenthumliche Arterienerkrankung(Periarteritisnodosa), diemitMorbusBrightii und rapid fortschreitenderallgemeiner-Muskelahmungeinhergeht. Deutsche Archiv KlinischeMedizin. 1866;1:484–518.
9. Leavitt RY, et al. The American College of Rheumatology 1990 criteria for the classification of Wegener’s granulomatosis. Arthritis Rheum. 1990;33:11017.
10. Jennette JC, et al. Nomenclature of systemic vasculitides. Proposal of an international consensus conference. Arthritis Rheum. 1994;37:187–92.
11. Yoshida M, et al. Report of clinical research subcommittee of small and medium-sized vessel vasculitis. Annual report of the subgroup for intractable vasculitis in the fiscal year of 1998, from research committee on specified immunological diseases, the Ministry of Health and Welfare of Japan. 1999, 239–46 (Japanese).
12. Koyama A, et al. A nationwide survey of rapidly progressive glomerulonephritis in Japan: etiology, prognosis and treatment diversity. Clin Exp Nephrol. 2009;13:633–50.
13. Sada KE, Amano M, et al. Research Committee on Intractable Vasculitides, the Ministry of Health, Labour, Welfare of Japan. A nationwide survey on the epidemiology and clinical features of eosinophilic granulomatosis with polyangiitis (Churg-Strauss) in Japan. Mod Rheumatol. 2014;24:640–4.
14. Watts R, et al. Development and validation of a consensus methodology for the classification of the ANCA—associated vasculitides and polyarteritis nodosa for epidemiological studies. Ann Rheum Dis. 2007;66:222–7.
15. Sada KE, Yamamura M, et al. Research Committee on Intractable Vasculitides, the Ministry of Health, Labour, Welfare of Japan. Issues associated with the Ministry of Health, Labour and Welfare diagnostic criteria for antineutrophil cytoplasmic antibody-associated vasculitides: Reclassification of patients in the associated vasculitides according to the MHLW criteria. Mod Rheumatol. 2015; 25:657–9.
16. Pankhurst T, et al. Malignancy is increased in ANCA—associated vasculitis. Rheumatology(Oxford). 2004;43:1532–5.
17. Karube M, et al. ANCA related vasculitis and malignant tumor. Annual Review Jinzo 2007.Chugai-Igaku-Sha, Tokyo, Japan. 2007;69–75 (Japanese).
18. Naicker S, et al. Infection and glomerulonephritis. Semin Immunopathol. 2007;29:397–414.
19. Iwata Y, et al. Shunt nephritis with positive titers for ANCA specific for proteinase 3. Am J Kidney Dis. 2004;43:e11–16.
20. Koyama A, et al. Glomerulonephritis associated with MRSA infection: a possible role of bacterial superantigen. Kidney Int. 1995;47:207–16.

III. Epidemiology and prognosis (incidence, prevalence, and outcome)

1. Endo M, et al. Estimates of the number of patients with four progressive renal diseases and epidemiological study on IgA nephropathy, report of progressive renal disease research 2004, research on intractable disease, the Ministry of Health, Labour and Welfare of Japan. 2005;163–7 (Japanese).
2. Watanabe T, et al. National epidemiological survey and application to the research on target number of the patient from DPC database. Report of progressive renal disease research 2011, research on intractable disease, the Ministry of Health, Labour and Welfare of Japan. 2012;53–62 (Japanese).
3. Sugiyama H, et al. Japan renal biopsy registry: the first nationwide, web-based, and prospective registry system of renal biopsies in Japan. Clin Exp Nephrol. 2011;15:493–503.
4. Yokoyama H, et al. Construction of Japan kidney disease registry and its analysis report of progressive renal disease research 2008–2010, research on intractable disease, the Ministry of Health, Labour and Welfare of Japan. 2011;17–22 (Japanese).
5. Current status of dialysis therapy in Japan as of Dec. 31st 2011. Committee of renal data registry, Japanese society for dialysis therapy. (http://www.jsdt.or.jp).
6. López-Gómez JM, et al. Spanish Registry of Glomerulonephritis. Renal biopsy findings in acute renal failure in the cohort of patients in the Spanish registry of glomerulonephritis. Clin J Am Soc Nephrol. 2008;3:674–81.
7. McQuarrie EP, et al. Centre variation in incidence, indication and diagnosis of adult native renal biopsy in Scotland. Nephrol Dial Transplant. 2009;24:1524–8.
8. Hedger N, et al. Incidence and outcome of pauci-immune rapidly progressive glomerulonephritis in Wessex, UK: a 10-year retrospective study. Nephrol Dial Transplant. 2000;15:1593–9.
9. Matsuo S, et al. Guidelines for the treatment of rapidly progressive glomerulonephritis, second version. Nihon Jinzo Gakkai Shi. 2011;53:509–55.
10. Koyama A, et al. A nationwide survey of rapidly progressive glomerulonephritis in Japan: etiology, prognosis and treatment diversity. Clin Exp Nephrol. 2009;13:633–50.
11. Day CJ, et al. Prediction of ESRD in pauci-immune necrotizing glomerulonephritis: quantitative histomorphometric assessment and serum creatine. Am J Kidney Dis. 2010;55:250–8.

IV. Treatment

1. Treatment algorithm

2. Clinical questions for treatment

CQ 1. Do the different ANCA assays influence the diagnostic assessment and disease activity evaluation in ANCA-associated vasculitis?

1. Hagen EC, et al. Kidney Int. 1998;53:743–53 (Level 4).
2. Csernok E, et al. Rheumatology (Oxford). 2004;43:174–80 (Level 4).
3. Holle JU, et al. Ann Rheum Dis. 2005;64:1773–9 (Level 4).
4. Trevisin M, et al. Am J Clin Pathol. 2008;129:42–53 (Level 4).
5. Ito-Ihara T, et al. Clin Exp Rheumatol. 2008;26:1027–33 (Level 4).

CQ 2. Do changes in ANCA levels in response to therapy predict disease relapse during the remission period of ANCA-associated vasculitis?

1. Han WK, et al. Kidney Int. 2003;63:1079–85 (Level 4).
2. Tomasson G, et al. Rheumatology (Oxford). 2012;51:100–9 (Level 1).

CQ 3. Is monitoring of anti-GBM antibody levels a useful tool to assess the disease activity and relapse in patients with anti-GBM nephritis and Goodpasture syndrome accompanied by RPGN?

1. Lockwood CM, et al. Lancet. 1976;(17962):711–5 (Level 5).
2. Johnson JP, et al. Am J Med. 1978;64:354–9 (Level 5).
3. Johnson JP, et al. Medicine (e Baltimore). 1985;64:219–27 (Level 2).
4. Yang R, et al. Nephrol Dial Transplant. 2009;24:1838–44 (Level 4).
5. Pedchenko V, et al. N Engl J Med. 2010;363:343–54 (Level 4).
6. Jia XY, et al. Clin J Am Soc Nephrol. 2012;7:926–33 (Level 4).
7. Herody M, et al. Clin Nephrol. 1993;40:249–55 (Level 4).
8. Levy JB, et al. Ann Intern Med. 2001;134:1033–42 (Level 4).
9. Cui Z, et al. Medicine (Baltimore). 2011;90:303–11 (Level 4).
10. Levy JB, et al. Am J Kidney Dis. 1996;27:573–8 (Level 5).
11. Kalluri R, et al. Transplantation. 2000;69:679–83.

CQ 4. Is renal biopsy useful in determining the treatment strategy for RPGN?

1. Bajema IM, et al. Kidney Int. 1999;56:1751–8 (Level 4).
2. Vergunst CE, et al. Am J Kidney Dis. 2003;41:532–8 (Level 4).
3. de Lind van Wijngaarden RA, et al. J Am Soc Nephrol. 2006;17:2264–74 (Level 2).
4. Day CJ, et al. Am J Kidney Dis. 2010;55:250–8 (Level 4).
5. Hauer HA, et al. Kidney Int. 2002;62:1732–42 (Level 4).
6. de Lind van Wijngaarden RA, et al. J Am Soc Nephrol. 2007;18:2189–97 (Level 2).
7. Berden AE, et al. J Am Soc Nephrol. 2010;21:1628–36 (Level 4).
8. Muso E, et al. Clin Exp Nephrol. 2013;17:659–62 (Level 4).
9. Pagnoux C, et al. Arthritis Rheum. 2010;62:616–26 (Level 4).
10. Inoue M, et al. Hum Pathol. 1998;29:223–7 (Level 5).
11. Bajema IM, et al. Nephrol Dial Transplant. 1996;11:1989–95 (Level 4).
12. Joh K, et al. Clin Exp Nephrol. 2008;12:277–91 (Level 4).
13. Johnson JP, et al. Medicine (e Baltimore). 1985;64:219–27 (Level 2).
14. Merkel F, et al. Nephrol Dial Transplant. 1994;9:372–6 (Level 4).
15. Levy JB, et al. Ann Intern Med. 2001;134:1033–42 (Level 4).
16. Walker RG, et al. Q J Med. 1985;54:75–89 (Level 4).

CQ 5. Is it recommended that the immunosuppressive treatment of anti-neutrophil cytoplasmic antibody (ANCA)-negative pauci-immune rapidly progressive glomerulonephritis (RPGN) be the same as that of ANCA-positive disease?

1. Hedger N, et al. Nephrol Dial Transplant. 2000;15:1593–9 (Level 4).
2. Chen M, et al. J Am Soc Nephrol. 2007;18:599–605 (Level 4).
3. Hauer HA, et al. Kidney Int. 2002;61:80–9 (Level 5).

CQ 6. Is it recommended that the treatment of PR3-ANCA-positive RPGN be the same as that of MPO-ANCA-positive disease?

1. Yamagata K, et al. Clin Exp Nephrol. 2012;16:580–8 (Level 4).
2. Harper L, et al. Rheumatology (Oxford). 2005;44:495–501 (Level 4).
3. Pagnoux C, et al. Arthritis Rheum. 2008;58:2908–18 (Level 4).

CQ 7. Should special care be given in the treatment of older patients with ANCA-associated RPGN compared with younger patients?

1. Harper L, et al. Rheumatology (Oxford). 2005;44:495–501.
2. Yamagata K, et al. Clin Exp Nephrol. 2012;16:580–8 (Level 4).

CQ 8. Is initial therapy with corticosteroids alone recommended for improving renal function and survival in patients with RPGN?

1. Frohnert PP, et al. Am J Med. 1967;43:8–14 (Level 5).
2. Bolton WK, et al. Am J Med. 1979;66:495–502 (Level 5).
3. Couser WG. Am J Nephrol. 1982;2:57–69 (Level 5).
4. Nachman PH, et al. J Am Soc Nephrol. 1996;7:33–9 (Level 3).
5. Hogan SL, et al. Ann Intern Med. 2005;143:621–31 (Level 4).
6. Adu D, et al. QJM 1997;90:401–9 (Level 2).
7. Lionaki S, et al. Kidney Int. 2009;76:644–51 (Level 4).
8. Bolton WK, et al. Am J Nephrol. 1989;9:368–75 (Level 4).
9. Hogan SL, et al. J Am Soc Nephrol. 1996;7:23–32 (Level 4).
10. de Lind van Wijngaarden RA, et al. J Am Soc Nephrol. 2006;17:2264–74 (Level 4).
11. Austin HA, et al. N Engl J Med. 1986;314:614–9 (Level 2).
12. Gourley MF, et al. Ann Intern Med. 1996;125:549–57 (Level 2).
13. Cui Z, et al. Medicine (Baltimore). 2011;90:303–11 (Level 4).
14. Levy JB, et al. Ann Intern Med. 2001;134:1033–42 (Level 4).
15. Johnson JP, et al. Medicin (e Baltimore). 1985;64:219–27 (Level 2).

CQ 9. Which of oral corticosteroid or intravenous pulse corticosteroid is recommended as an initial corticosteroid therapy for improving renal function and survival in patients with RPGN?

1. Adu D, et al. QJM. 1997;90:401–9 (Level 2).
2. Bolton WK, et al. Am J Nephrol. 1989;9:368–75 (Level 4).
3. Jayne DR, et al. J Am Soc Nephrol. 2007;18:2180–8 (Level 2).
4. Austin HA, et al. N Engl J Med. 1986;314:614–9 (Level 2).
5. Gourley MF, et al. Ann Intern Med. 1996;125:549–57 (Level 2).
6. Houssiau FA, et al. Arthritis Rheum. 2002;46:2121–31 (Level 2).
7. Mok CC, et al. Am J Kidney Dis. 2001;38:256–64 (Level 3).
8. Appel GB, et al. J Am Soc Nephrol. 2009;20:1103–12 (Level 2).
9. Levy JB, et al. Ann Intern Med. 2001;134:1033–42 (Level 4).
10. Johnson JP, et al. Medicine (e Baltimore). 1985;64:219–27 (Level 2).
11. Cui Z, et al. Medicine (Baltimore). 2011;90:303–11 (Level 4).

CQ 10. Is initial therapy with immunosuppressive agents recommended for improving renal function and survival in patients with RPGN?

1. Nachman PH, et al. J Am Soc Nephrol. 1996;7:33–9 (Level 3).
2. Hogan SL, et al. J Am Soc Nephrol. 1996;7:23–32 (Level 3).
3. De Groot K, et al. Arthritis Rheum. 2005;52:2461–9 (Level 2).
4. Steinberg AD, et al. Arthritis Rheum. 1991;34:945–50 (Level 2).
5. Houssiau FA, et al. Arthritis Rheum. 2002;46:2121–31 (Level 2).
6. Houssiau FA, et al. Ann Rheum Dis. 2010;69:61–4 (Level 2).
7. Chan TM, et al. N Engl J Med. 2000;343:1156–62 (Level 2).
8. Ginzler EM, et al. N Engl J Med. 2005;353:2219–28 (Level 2).
9. Kamanamool N, et al. Medicine (Baltimore). 2010;89:227–35 (Level 1).
10. Levy JB, et al. Ann Intern Med. 2001;134:1033–42 (Level 4).
11. Cui Z, et al. Medicine (Baltimore). 2011;90:303–11 (Level 4).

CQ 11. Which is recommended for improving renal and patient survival in RPGN, oral cyclophosphamide or intravenous pulses of cyclophosphamide?

1. de Groot K, et al. Ann Intern Med. 2009;150:670–80 (Level 2).
2. Adu D, et al. QJM 1997;90:401—9. (Level 2)
3. Guillevin L, et al. Arthritis Rheum. 1997;40:2187–98 (Level 2).
4. Haubitz M, et al. Arthritis Rheum. 1998;41:1835–44 (Level 2).
5. Walters GD, et al. BMC Nephrol. 2010;11:12 (Level 1).

CQ 12. Is immunosuppressive therapy recommended for improving renal function and survival in patients with RPGN who are receiving dialysis at the time of diagnosis?

1. Jayne DR, et al. J Am Soc Nephrol. 2007;18:2180–8 (Level 2).
2. Pepper RJ, et al. Clin J Am Soc Nephrol. 2013;8:219–24 (Level 4).
3. de Lind van Wijngaarden RA, et al. J Am Soc Nephrol. 2006;17:2264–74 (Level 2).
4. de Lind van Wijngaarden RA, et al. J Am Soc Nephrol. 2007;18:2189–97 (Level 2).
5. Kamanamool N, et al. Medicine (Baltimore). 2010;89:227–35 (Level 1).
6. Liang L, et al. J Rheumatol. 2004;31:701–6 (Level 4).
7. Hind C, et al. Lancet. 1983;(18319):263–5 (Level 4).
8. Levy JB, et al. Ann Intern Med. 2001;134:1033–42 (Level 4).
9. Flores JC, et al. Lancet. 1986;1(8471):5–8 (Level 5).

CQ 13. Is rituximab recommended for improving renal function and survival in patients with RPGN?

1. Specks U, et al. Arthritis Rheum. 2001;44:2836–40 (Level 5).
2. Keogh KA, et al. Am J Respir Crit Care Med. 2006;173:180–7 (Level 4).
3. Jones RB, et al. N Engl J Med. 2010;363:211–20 (Level 2).
4. Stone JH, et al. N Engl J Med. 2010;363:221–32 (Level 2).
5. Jones RB, et al. Arthritis Rheum. 2009;60:2156–68 (Level 4).
6. Berden AE, et al. J Am Soc Nephrol. 2012;23:313–21 (Level 4).
7. Specks U, et al. N Engl J Med. 2013;369:417–27 (Level 2).
8. Gregersen JW, et al. Scand J Rheumatol. 2013;42:207–10 (Level 4).
9. Mansfield N, et al. Nephrol Dial Transplant. 2011;26:3280–6 (Level 4).
10. Cartin-Ceba R, et al. Arthritis Rheum. 2012;64:3770–8 (Level 4).
11. Rhee EP, et al. Clin J Am Soc Nephrol. 2010;5:1394–400 (Level 4).
12. Díaz-Lagares C, et al. Autoimmun Rev. 2012;11:357–64 (Level 4).
13. Jónsdóttir T, et al. Rheumatology (Oxford). 2013;52:847–55 (Level 4).
14. Melander C, et al. Clin J Am Soc Nephrol. 2009;4:579–87 (Level 4).
15. Rovin BH, et al. Arthritis Rheum. 2012;64:1215–26 (Level 2).
16. Pepper R, et al. Nephrol Dial Transplant. 2009;24:3717–23 (Level 4).
17. Condon MB, et al. Ann Rheum Dis. 2013;72:1280–6 (Level 4).
18. Li EK, et al. Rheumatology (Oxford). 2009;48:892–8 (Level 2).
19. Syeda UA, et al. Semin Arthritis Rheum. 2013;42:567–72 (Level 5).

CQ 14. Is initial therapy with plasmapheresis recommended for improving renal function and survival in patients with RPGN?

1. Jayne DR, et al. J Am Soc Nephrol. 2007;18:2180–8 (Level 2).
2. Szpirt WM, et al. Nephrol Dial Transplant. 2011;26:206–13 (Level 2).
3. Walters GD, et al. BMC Nephrol. 2010;11:12 (Level 1).
4. Walsh M, et al. Am J Kidney Dis. 2011;57:566–74 (Level 1).
5. Yamagata K, et al. J Clin Apher. 2005;20:244–51 (Level 4).
6. Wei N, et al. Lancet. 1983;1(8314–5):17–22 (Level 2).
7. Lewis EJ, et al. N Engl J Med. 1992;326:1373–9 (Level 2).
8. Euler HH, et al. Arthritis Rheum. 1994;37:1784–94 (Level 5).
9. Yamaji K, et al. Ther Apher Dial. 2008;12:298–305 (Level 5).
10. Loo CY, et al. Transfus Apher Sci. 2010;43:335–40 (Level 2).
11. Cui Z, et al. Medicine (Baltimore). 2011;90:303–11 (Level 4).
12. Flores JC, et al. Lancet. 1986;1(8471):5–8 (Level 5).

CQ 15. Does anticoagulant or antiplatelet therapy improve mortality and morbidity in patients with RPGN?

1. Silfverskiöld BP, Scand Arch Physiol. 1940;175–82.
2. Kleinerman J, Lab Invest. 1954;3:495–508.
3. Vassalli P, et al. Am J Pathol. 1964;45:653–77.
4. Halpern B, et al. Nature. 1965;205:257–9.
5. Kincaid-Smith P, et al. Lancet. 1968;2(7583):1360–3 (Level 5).
6. Arieff AI, et al. Arch Intern Med. 1972;129:77–84 (Level 5).
7. Brown CB, et al. Lancet. 1974;2(7890):1166–72 (Level 5).
8. Fye KH, et al. Arch Intern Med. 1976;136:995–9 (Level 5).
9. Cunningham RJ, et al. Pediatr Res. 1980;14:128–32 (Level 5).
10. Hirahashi J, et al. Ann Intern Med. 2012;156:755–6 (Level 5).
11. Taji Y, et al. Clin Exp Nephrol. 2006;10:268–73.
12. Liu XJ, et al. Intern Med. 2011;50:2503–10.
13. Kessenbrock K, et al. Nat Med. 2009;15:623–5.
14. Hakkim A, et al. Proc Natl Acad Sci USA. 2010;107:9813–8.
15. Clark SR, et al. Nat Med. 2007;13:463–9.
16. Fuchs TA, et al. Proc Natl Acad Sci USA. 2010;107:15880–5.

CQ 16. Do intravenous immunoglobulins (IVIg) improve renal and patient survival in RPGN?

1. Martinez V, et al. French Vasculitis Study Group. Arthritis Rheum. 2008;58:308–17 (Level 3).
2. Jayne DR, et al. QJM. 2000;93:433–9 (Level 2).
3. Muso E, et al. Jpn J Infect Dis. 2004;57:S17–8 (Level 3).
4. Ito-Ihara T, et al. Nephron Clin Pract. 2006;102:c35–42 (Level 3).

CQ 17. Is maintenance therapy with corticosteroids alone recommended for improving renal function and survival in patients with RPGN?

1. Frohnert PP, et al. Am J Med. 1967;43:8–14 (Level 4).
2. Bolton WK, et al. Am J Med. 1979;66:495–502 (Level 4).
3. Couser WG, Am J Nephrol. 1982;2:57–69 (Level 5).
4. Bolton WK, et al. Am J Nephrol. 1989;9:368–75 (Level 4).
5. Nachman PH, et al. J Am Soc Nephrol. 1996;7:33–9 (Level 3).
6. Adu D, et al. QJM. 1997;90:401–9 (Level 2).
7. Walsh M, et al. Arthritis Care Res. 2010;62:1166–73 (Level 1).
8. Ozaki S, et al. Mod Rheumatol. 2012;22:394–404 (Level 4).
9. Austin HA, et al. N Engl J Med. 1986;314:614–9 (Level 2).
10. Gourley MF, et al. Ann Intern Med. 1996;125:549–57 (Level 2).
11. Houssiau FA, et al. Arthritis Rheum. 2002;46:2121–31 (Level 2).
12. Levy JB, et al. Ann Intern Med. 2001;134:1033–42 (Level 4).

CQ 18. What should be the reduction rate of oral corticosteroids?

1. Walsh M, et al. Arthritis Care Res. 2010;62:1166–73 (Level 1).
2. Jayne D, et al. N Engl J Med 2003;349:36–44 (Level 2).
3. Wada T, et al. J Rheumatol 2012;39:545–51 (Level 4).
4. Ozaki S, et al. Mod Rheumatol. 2012;22:394–404 (Level 4).

CQ 19. Is maintenance therapy with immunosuppressive agents recommended for improving renal function and survival in patients with RPGN?

1. Jayne D, et al. N Engl J Med. 2003;349:36–44 (Level 2).
2. Hirayama K, et al. Am J Kidney Dis. 2004;44:57–63 (Level 5).
3. Langford CA, et al. Arthritis Rheum. 1999;42:2666–73 (Level 4).
4. Langford CA, et al. Am J Med. 2003;114:463–9 (Level 4).
5. Hiemstra TF, et al. JAMA. 2010;304:2381–8 (Level 2).
6. Houssiau FA, et al. Ann Rheum Dis. 2010;69:2083–9 (Level 2).
7. Dooley MA, et al. N Engl J Med. 2011;365:1886–95 (Level 2).
8. Levy JB, et al. Ann Intern Med. 2001;134:1033–42 (Level 4).

CQ 20. Does trimethoprim/sulfamethoxazole improve renal prognosis and life prognosis?

1. Itabashi M, et al. Nephron Clin Pract. 2010;115:c21–c27 (Level 4).
2. Ozaki S, et al. Mod Rheumatol. 2012;22:394–404 (Level 3).
3. Reinhold-Keller E, et al. Arthritis Rheum. 2000;43:1021–32 (Level 4).
4. Ognibene FP, et al. Am J Respir Crit Care Med. 1995;151:795–9 (Level 4).
5. Guillevin L, et al. Arthritis Rheum. 1997;40:2187–98 (Level 2).
6. Green H, et al. Mayo Clin Proc. 2007;82:1052–9 (Level 1).
7. Stegeman CA, et al. N Engl J Med. 1996;4;335:16–20 (Level 2).
8. Delanaye P, et al. Nephron Clin Pract. 2011;119:c187–93 (Level 5).

